# Telehealth intervention involving the HEARTS Technical Package and the additional use of an activity monitor to increase physical activity level post-stroke: Protocol for a feasibility randomized controlled trial

**DOI:** 10.1371/journal.pone.0320026

**Published:** 2025-04-04

**Authors:** Paula da Cruz Peniche, Olive Lennon, Jordana de Paula Magalhães, Jéssica Melo dos Santos, Janaine Cunha Polese, Christina Danielli Coelho de Morais Faria

**Affiliations:** 1 Department of Physiotherapy, Universidade Federal de Minas Gerais, Belo Horizonte, Minas Gerais, Brazil; 2 School of Public Health, Physiotherapy and Sports Science, University College Dublin, Belfield, Dublin, Ireland; National Cerebral and Cardiovascular Center: Kokuritsu Junkankibyo Kenkyu Center, JAPAN

## Abstract

**Background:**

Low physical activity level is a common risk factor for recurrent stroke. Feasibility studies show behavior change interventions can increase physical activity participation, but face barriers (e.g., home visits or internet access). Low-cost telehealth approaches, like telephone calls, may overcome these challenges. Another low-cost strategy involves motivational tools supporting “Behavioral Regulation,” such as physical activity monitors. However, evidence is insufficient to support their use in increasing physical activity levels post-stroke. A systematic review suggests integrating these devices into multifaceted behavior change interventions (e.g., the 5As brief intervention outlined in the HEARTS Technical Package) may enhance their effectiveness. Combined with physical activity monitors and telephone follow-up, this approach has proven feasible for individuals post-stroke. These findings underscore the need to explore combining the 5As brief intervention with physical activity monitors to assess potential added benefits. This feasibility randomized controlled trial (RCT) study will investigate whether the telehealth intervention (by telephone call) combining the 5As brief intervention, as outlined in the HEARTS Technical Package, and physical activity monitoring, compared to a control group receiving only the 5As brief intervention, is feasible and supports a fully powered RCT.

**Methods:**

A feasibility RCT study, with blinded assessment, will assign 24 individuals post-stroke (diagnosed ≥ 6 months), aged ≥ 18 years, inactive, able to walk 10 meters independently, and medically approved for physical activity, to experimental (n = 12) or control group (n = 12). Both groups will undergo the 5As brief intervention (Ask, Advise, Assess, and Assist delivered face-to-face, and Arrange via telephone call follow-up), for 12 weeks, with the experimental group also using a physical activity monitor. Outcomes include feasibility of recruitment, intervention, measurement, and blinding the outcome assessor, cost and clinical outcomes.

**Discussion:**

The intervention aligns with stroke secondary prevention recommendations and utilizes low-cost telehealth approaches. This study will contribute to defining future RCT phases.

**Trial registration:**

ClinicalTrials.gov NCT06068036.

## Introduction

Recurrent strokes, although largely preventable, contribute significantly to the high global stroke burden [1,2]. They can be prevented using evidence-based strategies emphasizing lifestyle modifications to manage modifiable risk factors [1,3]. A low level of physical activity is a key modifiable risk factor for recurrent stroke [4–9]. Physical inactivity is commonly observed in individuals post-stroke [4–9] and is associated with an increased risk of stroke recurrence, mortality, and other cardiovascular conditions (e.g., heart attack) [8,9]. Additionally, physical inactivity is associated with depressive symptoms, poor sleep quality, and a reduced quality of life [10]. Therefore, addressing this modifiable risk factor - low physical activity levels - is crucial by encouraging participation in physical activity among individuals post-stroke.

Encouraging physical activity participation post-stroke is complex and requires personalized interventions focused on lifestyle behavior change [4]. Such interventions must include educational strategies, be theoretically-informed, and be tailored to individual needs [9,11,12]. It is also crucial to consider factors such as personal preferences, environment context, available resources [9,11,12], barriers (e.g., cost and lack of information on how to exercise), and facilitators (e.g., beliefs about health improvement and reducing the risk of recurrent stroke) [13]. Intervention planning should also be guided by established guidelines on the frequency, intensity, and duration of physical activity post-stroke [4]. Recent feasibility studies promoting physical activity participation with individuals post-stroke have shown that interventions meeting these criteria, delivered both in person [14,15] and via telehealth [16], are feasible and can increase participation. However, challenges such as the need for home visits [14,15] or internet access [16] limit their clinical applicability. Implementing low-cost telehealth interventions, such as telephone calls, could help address these barriers and enhance access to support.

Another low-cost approach to encouraging physical activity participation among individuals post-stroke involves the use of physical activity monitors [17,18]. These devices are considered motivational tools for promoting physical activity engagement [19–22], aligning with “Behavioral regulation”, a construct within the Theoretical Domains Framework (TDF) of behavior change, which supports self-monitoring by tracking steps [23]. The use of physical activity monitors is also highlighted in key guidelines for promoting physical activity post-stroke [4]. However, a systematic review investigating the impact of these devices on increasing physical activity levels in individuals post-stroke concluded that the evidence remains insufficient to support their use in clinical practice [17]. Notably, the evidence in the systematic review primarily stemmed from studies conducted in hospital settings (three of the four included studies) [17]. Sedentary lifestyle, a significant influence on physical activity levels post-stroke [6], is challenging to modify within the constraints of hospital settings. The review’s authors reported very little and limited confidence in the estimated effects of the analyzed outcomes [17]. Moreover, the authors hypothesized that integrating physical activity monitors into a multifaceted behavior change intervention might prove more effective in increasing physical activity levels post-stroke in future studies [17].

One such multifaceted behavior change intervention is the 5As brief intervention (Ask, Advise, Assess, Assist, and Arrange), outlined in the HEARTS Technical Package [24]. Developed by organizations such as the World Health Organization (WHO) and the World Stroke Organization (WSO), the HEARTS Technical Package is an established and recommended approach for improving cardiovascular health [24]. It comprises six modules: H – “Healthy lifestyle,” E – “Evidence,” A – “Access,” R – “Risk,” T – “Team,” and S – “Systems for monitoring” [24]. The H – “Healthy lifestyle” module outlines the 5As brief intervention for promoting physical activity [24]. This intervention involves planning, practical counseling, and social support [24], addressing seven theoretical domain of behavior change: “Knowledge”, “Intentions”, “Beliefs about capabilities”, “Goals”, “Social influences”, “Environmental context and resources”, and “Reinforcement” [23]. The 5As brief intervention has been implemented with individuals post-stroke [25–27] and in other populations (e.g., diabetics, hypertensives and obese individuals) [28–30] involving the encouragement of the adoption of healthy lifestyle behaviors. Additionally, studies on cardiovascular risk management [31,32] found the HEARTS Technical Package [24] feasible for implementation by communities, patients, providers, decision-makers, and funders. However, current evidence has yet to integrate this multifaceted behavior change intervention with the use of physical activity monitors to demonstrate the additional effect of this technology on increasing physical activity levels in individuals post-stroke.

Recently, Liljehult et al. [27] demonstrated that the 5As brief intervention, combined with the use of physical activity monitors and follow-up via telephone, is feasible for implementation with individuals post-stroke. However, in this study [27], the control group received usual care, making it impossible to determine the additional effect of incorporating physical activity monitors into the multifaceted behavior change intervention. Therefore, the aims of this feasibility randomized controlled trial (RCT) study are 1. to investigate whether the telehealth intervention (by telephone call) combining the 5As brief intervention, as outlined in the HEARTS Technical Package [24], with the use of a physical activity monitor, compared to a control group receiving only the 5As brief intervention (by telephone call), is feasible to be implemented and 2. to estimate the parameters for conducting a fully powered RCT.

## Materials and methods

### Design

**Fig 1:**
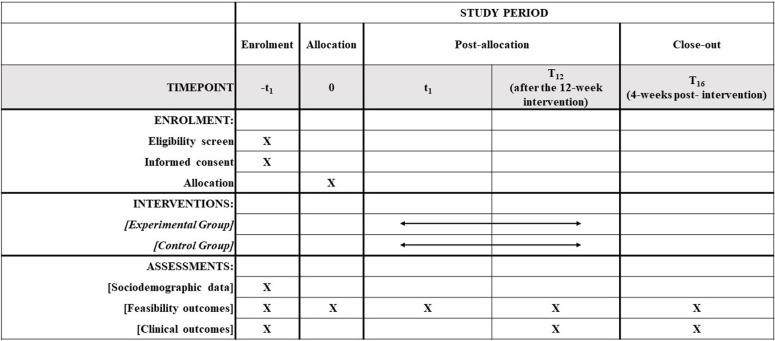
Schedule of enrolment, interventions, and assessments.

**Fig 2:**
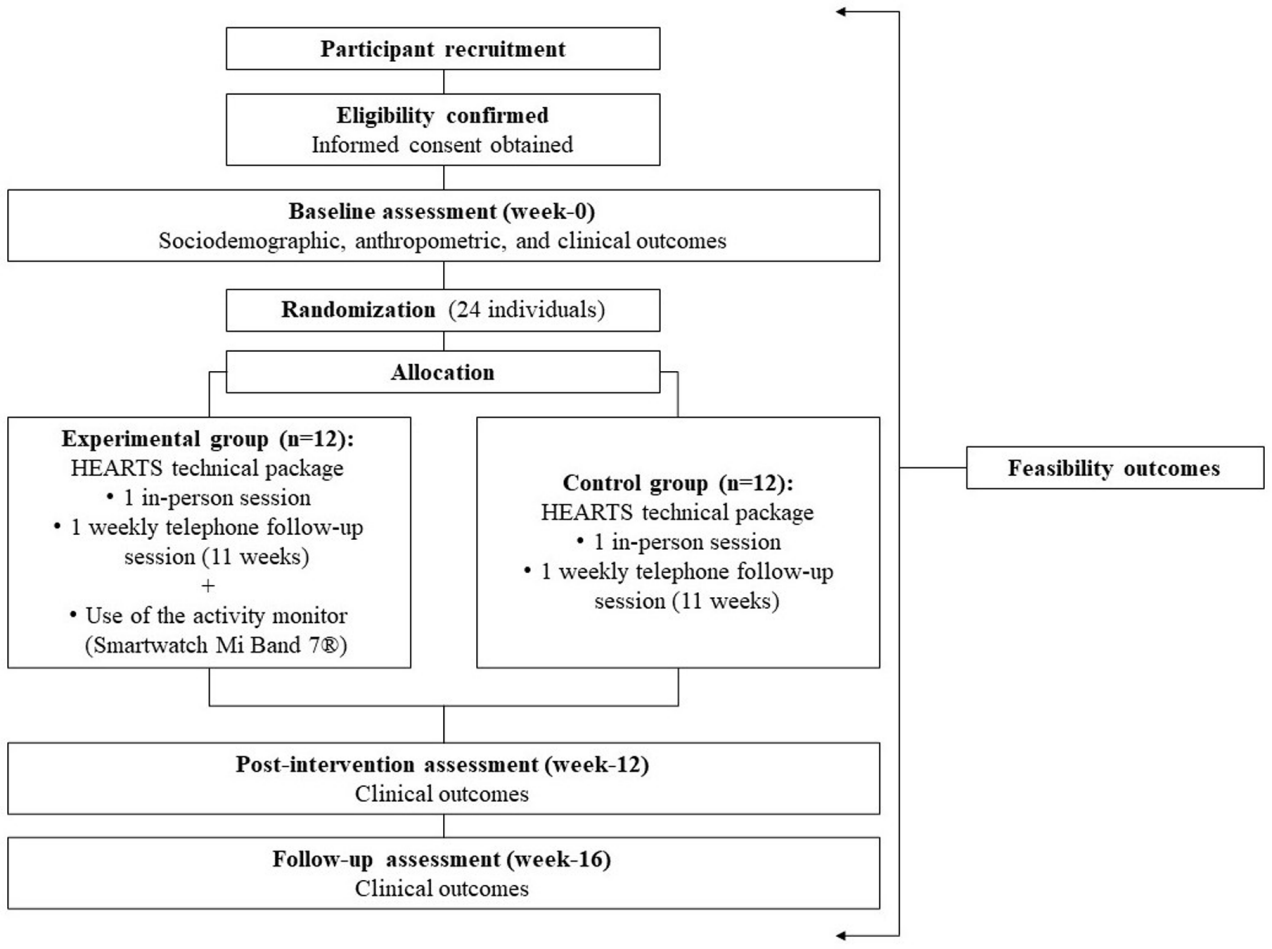
Study conduction flowchart.

**Fig 3:**
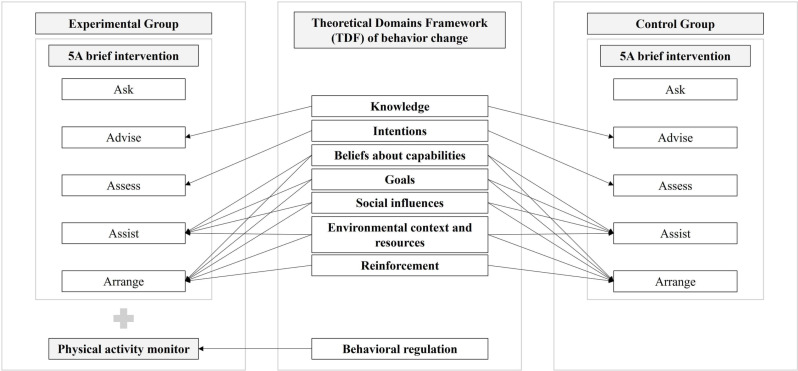
Mapping of the telehealth intervention proposed in the present study considering the Theoretical Domains Framework (TDF) of behavior change proposed by Atkins et al. [ 23].

This phase 1 feasibility RCT study [33,34], with concealed allocation and blinded assessments, that will adhere to the CONSORT extension for pilot or feasibility trials [34] will be carried out in Belo Horizonte (Minas Gerais, Brazil). This study was prospectively registered with ClinicalTrials.gov (NCT06068036) and received approval from the institutional ethics review board (Comitê de Ética em Pesquisa, Universidade Federal de Minas Gerais, CAAE: 66980723.2.0000.5149, number: 5.980.632, April 3, 2023). Fig 1 shows the schedule of enrolment, interventions, and assessments. Fig 2 shows the study conduction flowchart. Community-dwelling individuals post-stroke will be recruited and randomly assigned to experimental group or control group (Fig 3). Participant recruitment began in October 2023, and is expected to be completed in October 2024.

### Participants

A non-probabilistic sample will be recruited from the community. Recruitment strategies will include [35]:

1. Circulation of physical leaflets: these leaflets will be distributed in strategic locations in the city (e.g., universities, hospitals, health centers, outpatient clinics, rehabilitation clinics, and churches);
2. Referral by other researchers and health professionals: researchers and health professionals in the city will be contacted via conversations, email, social media, and phone calls, will be informed of the study, and will be invited to publicize the study or provide contact details of individuals post-stroke who could participate in the study. Physical and/or digital leaflets will be provided to these researchers/professionals.
3. Targeted campaign in support groups: support groups for individuals post-stroke in the city will be identified, contacted, and invited to publicize the study or provide contact details of individuals post-stroke who could participate in the study. Physical and/or digital leaflets will be provided.
4. Online advertising: attempts will be made to publicize the study on websites (e.g., university website); a specific social media to publicize the study will be created; and social media of organizations and institutions (e.g., other universities) will be identified and invited to publicize the study.

Individuals will be included according to the following criteria: present with a medical diagnosis of stroke and in the chronic phase (≥6 months); be aged ≥ 18 years; be classified as “Inactive” according to Adjusted Activity Score (AAS) from the Human Activity Profile (HAP) [36]; be able to walk 10 meters independently with or without a walking device (considering that walking is a preferred physical activity pointed by individuals post-stroke [37,38] and is a potential way to participate in physical activity [4]; and provide medical approval to participate in physical activity [39]. Individuals will be excluded according to the following criteria: present with a positive screening test for possible cognitive impairment determined by the education-adjusted cut-off scores (in points) of the Mini-Mental State Examination (illiterate: 13 points; elementary and middle school: 18 points; and high-school: 26 points) [40]; and have pain or other disorders (e.g., vestibular disorders, severe arthritis or any other diagnosed disease of the nervous system) precluding their participation [39].

### Randomization

A blinded trained researcher, not involved in the study, will generate the computer-randomization sequence prior to study commencement and will store the sequential numbers in opaque sealed envelopes. Individuals will be randomly allocated (1:1) to experimental or control group (Figs 2 and 3) by another blinded trained researcher. After confirmation of eligibility, the envelopes containing the random allocation sequence will be revealed by the physiotherapist researcher responsible for implementing the intervention.

### Intervention

Both groups will receive the telehealth intervention based on the 5As brief intervention (Ask, Advise, Assess, Assist, Arrange) to promote physical activity participation presented in the HEARTS Technical [24] (Fig 3). This established and recommended behavior change theoretically-informed telehealth intervention aligns with TDF constructs for behavior change (Fig 3) [23]. The first A (Ask) involves asking the participants about their physical activity participation in the last week [24]. The second A (Advise) involves advising the participant (in a clear, simple and personalized way) about participation in physical activity [24]. The researcher will inform the participants about recommended time for participation in physical activity per week, and advantages related to this health behavior [24], which aligns with the TDF construct for behavior change “Knowledge” [23]. The third A (Assess) involves assessing the participant’s readiness to initiate behavior change to become physically active and whether the individual believes they will be successful in increasing physical activity levels [24]. This part aligns with the TDF construct for behavior change “Intentions”, which refers to a conscious decision to perform a behavior [23], such as becoming physically active. The fourth A (Assist) involves helping the participant devise a plan that increases the chances of successfully increasing participation in physical activity, and providing practical counselling [24]. In this section, the researcher will help the participants to identify parts of their daily life where they could start to increase their activity levels, to identify activities that they would enjoy doing, and to identify possible challenges and suggest how to overcome them [24]. Therefore, these aspects are aligned with the TDF construct for behavior change “Beliefs about capabilities”, where the participants will be able to modify their self-confidence and perceived competence to become physically active [23], In addition, the cooperation between the participants and the researcher to develop the plan aligns with the TDF construct for behavior change “Goals”, where they will set goals to increase their participation in physical activity [23]. This part also aligns with the TDF construct for behavior change “Social influences”, where the researcher will provide social support [23]. Finally, the activities involved in this step also align with the TDF construct for behavior change “Environmental context and resources”, where factors related to the participants’ environment will be considered to encourage their participation in physical activity [23]. The fifth A (Arrange) involves following up with the participant, which can be by telephone, to discuss successes (and congratulate them) and difficulties (discuss ways to face challenges and encourage renewed commitment to the plan) [24]. Therefore, this part aligns with the TDF construct for behavior change “Reinforcement” [23], since these activities have the objective to increase the chance that the individual will continue to want to become physically active. As this last step involves revising the plan and the social support provided by the researcher, the others four TDF constructs for behavior change (“Beliefs about capabilities”, “Goals”, “Social influences”, “Environmental context and resources”) [23] mentioned before are also aligned to this part.

Participants will undertake a 12-week intervention. A researcher will be responsible for implementing the intervention. She has experience in conducting research with individuals post-stroke in-person and remotely, including conducting activities that aimed to encourage the adoption of healthy lifestyle behaviors in the context of stroke secondary prevention. In addition, she has completed training focused on the implementation of the proposed intervention. In week one, participants will complete the first four steps of the 5As brief intervention (Ask, Advise, Assess, Assist), following the script provided in the HEARTS Technical Package [24] in-person, in a university setting, with the physiotherapist researcher mentioned above. They will receive the diary of regular participation in physical activity, will be guided on how to fill it out, and will schedule the best times to receive the weekly telephone call [23]. Also following the script provided in the HEARTS Technical Package [24], the fifth A (Arrange) will be carried out for the subsequent 11 weeks, where participants in their home setting will be followed up remotely, by the same physiotherapist and by telephone call [24], once a week. If the participant does not answer the telephone call on the previously defined day and time, two attempts at new calls on different days will be made.

### Experimental group

Participants will undergo the telehealth intervention involving the 5As brief intervention (Ask, Advise, Assess, Assist), following the script provided in the HEARTS Technical Package [24], and the use of an activity monitor (Smartwatch Mi Band 7®) (Fig 3). The use of this physical activity monitor fits into the TDF construct for behavior change “Behavioral regulation”, where the participants can objectively observe their physical activity practice (self-monitor) and influence their decision about this behavior [23], Participants will receive their physical activity monitor, will be advised on how to use it, and will be instructed to return it at the end of the intervention (week-12). Physical activity monitors from the same brand have been used in other clinical trials [41–44]. The Smartwatch Mi Band 7® model was obtained for this study and the cost was approximately 50$ (R$235) each.

### Control group

Participants will perform the telehealth intervention involving the 5As brief intervention (Ask, Advise, Assess, Assist), following the script provided in the HEARTS Technical Package [24] (Fig 3). These participants will not receive the physical activity monitor.

### Procedures

All subjects will be informed about the study procedures and will provide written consent. The feasibility outcomes will be identified considering the different phases of conducting the study (Figs 1 and 2). A trained researcher, blinded to the group allocation, will collect the sociodemographic, anthropometric, and clinical outcomes, in a university laboratory setting. Two independent examiners, blinded to the group allocation, will enter the data into a statistical software package, verify any missing or apparently wrong values. Original paper forms will be kept in a secure place. Electronic files will be available only to the research team. Analysis will be carried-out by a researcher, blinded to the group allocation. All individuals will receive an identification code to ensure anonymity.

### Outcome of interest

#### Feasibility of recruitment.

Feasibility of recruitment will be determined by the ratio between the total number of eligible individuals (EI) and the total number of screened individuals (SI) (measure = EI/IS), and by the ratio between the total number of eligible individuals (EI) and the total number of recruited individuals (RI) (measure = EI/RI) [15,35,45–47]. In addition, the time expenditure for recruitment of study participants will also be identified [48]. A measure of EI/IS ≥ 10% will be considered as a success criterion [45].

#### Feasibility of intervention.

Feasibility of intervention will be determined by examining retention, follow-up of individuals, attendance, safety, satisfaction with treatment and perceived effectiveness.

Retention will be determined by the ratio between the total number of individuals who completed the proposed intervention program and the total number of individuals who started the proposed intervention program. The reasons for individuals dropping out of the intervention program will be recorded [35,45–47,49–51]. A measure of ≥ 90% retention will be considered as a success criterion [45].

Follow-up of individuals will be determined by the ratio between the total number of individuals who remained in the same group to which they were initially allocated until follow-up and the total number of individuals who were allocated [47,51].

Attendance will be determined by the ratio between the total number of sessions performed and the total number of sessions offered. The number and reasons for absence for sessions will be recorded [15,35,45,47]. A measure of ≥ 85% completion of in home proposed 11 sessions will be considered as a success criterion [45].

Safety will be determined by the number and reasons of adverse events (e.g., pain, falls, hospitalization, and death) identified during the period of intervention and follow-up of the individual [15,45,47,49,50].

Satisfaction with treatment will be assessed by the following situation: “Please listen to the sentence I am about to say and select the answer that is closest to your opinion. There is no right or wrong answer, we are interested in your opinion. “Overall, I am satisfied with the type of treatment I received”: totally agree, partially agree, agree, neither agree nor disagree, disagree, partially disagree, totally disagree”. The response provided will be registered by the examiner. A measure of ≥ 90% of participants reporting “totally agree, partially agree, or agree” will be considered as a success criterion [45].

Perceived effectiveness will be determined by the following question: “Comparing how it was before you carried out the intervention and now, do you think that your ability to perform routine physical activity is: much worse, moderately worse, a little worse, the same, a little better, moderately better, or much better?”. The response provided will be registered by the examiner.

#### Feasibility of measurement.

Feasibility of measurement will be determined by the percentage of clinical outcomes measured (ratio between the number of clinical outcomes measured and the number of clinical outcomes proposed to be measured), and the percentage of participants who filled-out the diary (ratio between the number of individuals who filled-out the diary and the number of individuals who started the proposed intervention program) [15]. The diary will be considered completed if participants provide information regarding physical activity practice for at least 75% (8 weeks) of the 11 weeks of intervention proposed to be carried out remotely [52]. The time taken to obtain the outcome measures will be recorded. In addition, the number and reasons of adverse events (e.g., pain, falls, hospitalization, and death) identified during the assessments will be registered.

#### Feasibility of blinding the outcome assessor.

The feasibility of blinding the outcome assessor will be determined by the number of participants and percentage of treatment allocations correctly guessed by the blinded assessor [53].

#### Cost.

Costs for staff training (e.g., materials and transportation), recruitment (e.g., related to telephone call and printing of folders), screening, examination of the inclusion and exclusion criteria and investigation of the outcomes (e.g., transportation for both participants and staff, printing of evaluations, and material costs), as well as the execution of the intervention (e.g., related to telephone call) will be registered [54].

#### Clinical outcomes.

Clinical outcomes will be evaluated to estimate the parameters for conducting a fully powered RCT including: physical activity levels and the number of individuals post-stroke who met the threshold to be considered physically active, systolic blood pressure (SBP) and diastolic blood pressure (DBP), cardiorespiratory fitness, self-efficacy for physical activity and health-related quality of life.

Physical activity levels and the number of individuals post-stroke who became more physically active will be measured with the HAP [36]. The questionnaire has 94 items hierarchically graded according to metabolic equivalent [36]. AAS, one of the HAP measures, provides a best estimate of the respondent’s average level of energy expended [36]. Higher scores indicate better outcomes [36]. Another measure of the HAP is the “Activity Rating”, which provides an overall rating of the respondent’s activity level into “Inactive”, “Moderately active” and “Active” [36]. The mean difference between groups and the standard deviation for the AAS outcome will be used to estimate the required sample size to conduct a future fully powered RCT.

SBP and DBP will be assessed by a trained researcher using Tycos® aneroid sphygmomanometer (WelchAllyn Inc., NY, USA, Model DS44) and stethoscope (Litmann Classic II SE 3M®, USA) and following these steps: the participants will be seated at rest in a chair, with legs uncrossed, for 5–10 minutes prior to the evaluation commencing; the sphygmomanometer will be positioned on the non-hemiplegic arm of the participant, and their arm will be supported at the level of the heart [55,56]. These measures will be operationalized in millimeters of mercury (mmHg).

Cardiorespiratory fitness will be measured by HAP [36,57]. One of the HAP measures is “Lifestyle Energy Consumption” (LEC), operationalized in ml.kg^-1^.min^-1^. [36]. The HAP is a valid instrument to estimate the cardiorespiratory fitness of individuals post-stroke [57].

Self-efficacy for physical activity will be measured by the Self-efficacy for Physical Activity Scale, and will be operationalized as the total score [15,58]. The scale has 10 items and has two response options: “yes” is computed as 1 and “no” is computed as 0 [58]. The total score is defined by the sum of the scores for each item [58]. Therefore, the minimum total score is 0 and the maximum total score is 10 [58]. Higher scores indicate better outcomes [58]. The Self-efficacy for Physical Activity Scale presents adequate validity, internal consistency, and reliability [58].

Health-related quality of life will be measured by the Stroke-Specific Quality of Life Scale (SSQOL), and will be operationalized as the total score [59]. The instrument has 49 items distributed across 12 domains [59]. The instrument has three different sets of responses and the items are rated on a 5-point Likert scale. The total score is defined by the sum of the scores for each item [59]. Therefore, the minimum total score is 49 and the maximum total score is 245 [59]. Higher scores indicate better outcome [59]. SSQOL presents adequate measurements properties when applied in individuals post-stroke [59].

### Sample size calculation

As a feasibility study, no formal sample size calculation is conducted [60]. Following the recommendation proposed by Julious [61] for feasibility studies with parallel groups, the present feasibility RCT study will present a sample size of 24 individuals, with 12 individuals in each group.

### Statistical analyzes

All analyses will be performed using SPSS version 21.0 (SPSS Inc., Chicago, IL, USA). Descriptive statistics will be calculated for all outcomes. The mean difference between groups and the standard deviation for the AAS outcome obtained from the HAP [35] will be identified. No studies investigating the minimal detectable change (MDC) (or related measures) for this outcome in individuals post-stroke were identified to date. However, Davidson et al. [62], in a systematic review, calculated the MDC at 90% confidence for the AAS obtained by the HAP in individuals with chronic conditions (e.g., chronic pain and arthritis) and reported a value of 6.8. A change greater than 6.8 for the AAS is expected in the present study. The effect sizes for this outcome will be calculated to determine the magnitude of within-groups and between-groups comparisons. The level of significance will be set at 5%.

### Ethics

This phase 1 feasibility RCT study [34] was prospectively registered with ClinicalTrials.gov (NCT06068036) and received approval from the institutional ethics review board (Comitê de Ética em Pesquisa, Universidade Federal de Minas Gerais, CAAE: 66980723.2.0000.5149, number: 5.980.632, April 3, 2023). Participants will provide signed, informed consent form. Researchers will take appropriate measures to ensure privacy, confidentiality, and data protection. This protocol will not generate primary data. However, data collected during the conduct of the trial will be made available in an open data repository.

## Discussion

To the best of our knowledge, this will be the first study to investigate whether the telehealth intervention (provided by telephone call) utilizing the 5As brief intervention, as outlined in the HEARTS Technical Package [24], alongside provision of an activity monitor, compared to a control group receiving only the 5As brief intervention (by telephone call), is feasible to be implemented. The results of the present study will also inform a fully powered RCT.

Insufficient evidence to recommend the use of physical activity monitors to increase physical activity levels post-stroke is based primarily on studies conducted in the hospital setting [17]. Considering that changing physical activity levels involves modifying sedentary lifestyle behaviors [6], the use of physical activity monitors in the real-world individual post-stroke setting has the potential to result in an additional change in physical activity levels post-stroke. The need for further studies investigating the additional effect of physical activity monitors integrated with multifaceted behavior change interventions to increase the physical activity level in individuals post-stroke has been highlighted in the literature [17]. The 5As brief intervention, a multifaceted behavior change approach, combined with the use of physical activity monitors and follow-up via telephone call, has been shown to be feasible for implementation with individuals post-stroke [27]. Based on this, the present study compared this intervention with a control group receiving only the 5As brief intervention, also delivered via telephone call.

Studies aiming to increase physical activity participation post-stroke using theoretically-informed behavior change interventions have included individuals in the acute phase [14], the subacute phase [15] or from the acute to the chronic phase [16]. In the present study, individuals post-stroke in the chronic phase will be included. The interventions offered in these previous studies have limited clinical applicability, as they require home visits [14,15] or internet access [16]. In contrast, the intervention in the present study will be delivered via telehealth, using low-cost technologies (e.g., telephone call). This approach is expected to overcome these barriers, enhancing the clinical applicability of the intervention.

Finally, the presented intervention - the 5As brief intervention outlined in the HEARTS Technical Package [24], combined with the use of a physical activity monitor - is characterized as a multifaceted theoretically-informed behavior change intervention, as it comprises several constructs from the TDF for behavior change [23] (Fig 3), meeting the stroke secondary prevention recommendations [8,9]. Moreover, the additional use of the physical activity monitor introduces a theoretical domain (“Behavioral regulation”) not addressed in the 5As brief intervention, supporting the rationale for its potential to produce an additional effect in increasing physical activity levels. To evaluate this, and in line with recommendations for conducting a RCT, it is necessary to first conduct this phase 1 feasibility RCT study [34].

A limitation of the present study is that the results will not be able to be generalized for individuals post-stroke who are not able to walk independently. Therefore, future studies aiming to promote participation in physical activity using the proposed intervention should address this topic. Furthermore, although the sample size of the present study can be considered as small, it should be taken into account that it was determined considering recommendations from the literature for feasibility studies [60,61]. Related to this last point, it should be noted that the main objective of the present study is not to investigate the effectiveness of the proposed interventions, since the feasibility study does not have this objective [63]. The preliminary analysis of clinical outcomes will focus on effect sizes as signs of improvement (or not) that justify planning to conduct a definitive fully powered RCT.

## Supporting information

S1 File Complete protocol approved by the ethics committee (in English). (PDF)

S2 File Complete protocol approved by the ethics committee (in Portuguese). (PDF)

S3 File SPIRIT checklist. (DOC)
