# Supplementary material for: Telehealth intervention involving the HEARTS Technical Package and the additional use of an activity monitor to increase physical activity level post-stroke: Protocol for a feasibility randomized controlled trial
Source: PLoS One. 2025 Apr 4;20(4):e0320026. doi: 10.1371/journal.pone.0320026 (PMC11970671; doi:10.1371/journal.pone.0320026)
Supplement: S1 File — (PDF) [file pone.0320026.s001.pdf]

IMPLEMENTATION OF A THEORETICALLY-INFORMED INTERVENTION BY TELEHEALTH TO INCREASE THE LEVEL OF PHYSICAL ACTIVITY OF INDIVIDUALS POST-STROKE: FEASIBILITY OF A RANDOMIZED CLINICAL TRIAL

Coordinating researcher: Professor Christina Danielli Coelho de Morais Faria, PhD, active professor at the Universidade Federal de Minas Gerais (UFMG), with a permanent contract, at the Escola de Educação Física, Fisioterapia e Terapia Ocupacional, Department of Physiotherapy.

Area of knowledge: Health Sciences

Belo Horizonte

Universidade Federal de Minas Gerais

Escola de Educação Física, Fisioterapia e Terapia Ocupacional

Department of Physiotherapy

2023

1. INTRODUCTION

Stroke is a health condition that has a high burden throughout the world, especially in underdeveloped and developing countries, such as Brazil (Feigin et al., 2021). This high burden is associated with the high incidence and prevalence of this health condition and the important disabilities that compromise the health and well-being of individuals (Feigin et al., 2021, Damsbo et al., 2020, Carvalho-Pinto et al., 2016). More than 12 million new cases of stroke occur annually, and it has been estimated that one in four people over the age of twenty-five will suffer an episode of stroke during their lifetime (Feigin et al., 2022). Currently, there are more than 100 million people surviving strokes in the world, 67% of whom are under 70 years of age (Feigin et al., 2022). Furthermore, it has been identified that, annually, more than 143 million years of healthy life are lost due to stroke-related deaths and disabilities (Feigin et al., 2022).

Another contributing factor to the high burden of stroke worldwide is its recurrence (Feigin et al., 2021, Lin et al., 2021). Recurrent stroke has a high incidence and prevalence, is considered the main cause of hospital readmissions during the first year after the stroke episode, results in an even more severe disability, and consequently, more impaired health, well-being and quality of life when compared to individuals who suffered an episode of stroke, and contributes to an increase of approximately 40% in healthcare costs (Lin et al., 2021, Wang et al., 2016, Zhong et al., 2016, Modrego et al., 2000). These facts point to an important public health need: actions for stroke secondary prevention. This need has been identified by the Stroke Association and James Lind Alliance, which identified stroke secondary prevention as one of the top 10 priority research areas to reduce the burden of stroke (Stroke Association and James Lind Alliance, 2021). Furthermore, this priority became even more evident in the context of the Corona Virus Disease-19 (COVID-19) pandemic, which compromised the rehabilitation process of individuals post-stroke (Cadilhac et al., 2021).

Guidelines state that the rehabilitation process for individuals post-stroke should aim to keep them well and free from additional events (Gittler et al., 2018, Winstein et al., 2016). These guidelines also highlight the importance of including interventions aimed at adopting a healthy lifestyle in stroke secondary prevention programs (Gittler et al., 2018, Winstein et al., 2016). Specific guidelines on stroke secondary prevention, in addition to guiding the adoption of a healthy lifestyle, also recommend the implementation of multimodal intervention programs (Gladstone et al., 2021, Kleindorfer et al., 2021). Multimodal intervention programs may include, for example, health

education actions related to medication adherence, knowledge about stroke and risk factors for the occurrence of stroke, and increasing the practice of physical activity (Lawrence et al. , 2015). However, despite recent guidelines and recommendations, the implementation of actions for stroke secondary prevention is still infrequent.

Recently, the concept of stroke secondary prevention based on non-surgical and non-pharmacological interventions was defined using the Delphi method (Lawrence et al., 2019). This definition addresses improving health and well-being, as well as reducing the risk of recurrent stroke, based on the implementation of theoretically-informed interventions and educational strategies (Lawrence et al., 2019). Furthermore, the concept presented guides that interventions aimed at managing risk factors related to lifestyle must be contextualized and individualized considering the capabilities, needs and priorities of individuals, as well as their families (Lawrence et al., 2019). Managing the set of risk factors related to lifestyle, such as smoking, harmful alcohol consumption, inadequate diet and physical inactivity, has been considered the cornerstone of stroke secondary prevention strategies (Gladstone et al., 2021, Kleindorfer et al., 2021, PAHO, 2020).

Management of this set of risk factors has been considered the cornerstone of stroke secondary prevention strategies because behavioral risk factors represent approximately 47% of the stroke burden (Feigin et al., 2022). In addition to the direct benefits achieved by its management, indirect benefits related to the management of cardiovascular risk factors can also be identified (Gladstone et al., 2021, Kleindorfer et al., 2021, PAHO, 2020). Cardiovascular risk factors include, for example, high blood pressure, dyslipidemia, hypercholesterolemia and diabetes (Feigin et al., 2022, Gladstone et al., 2021, Kleindorfer et al., 2021, PAHO, 2020). However, to better target the implementation of actions for stroke secondary prevention, it is important to consider, as previously pointed out, that these actions must be contextualized and individualized considering the capabilities, needs and priorities of individuals, as well as their families (Lawrence et al ., 2019).

There are still few studies that detail the Brazilian context regarding the profile of adoption of healthy lifestyle behaviors related to the pillar of stroke secondary prevention strategies: smoking cessation, safe alcohol consumption, adequate diet and regular physical activity. Ribeiro et al. (2012), in one of the few studies developed on this topic in Brazil, showed that, of the 140 individuals post-stroke living in João Pessoa-PB included in the study, 79% did not consume alcoholic beverages and 84% were not smokers. Trabaquini (2015) showed that, of the 30 individuals post-stroke living in

Andirá-PR included in the study, 70% never smoked, 17% stopped smoking and 13% were smokers; 60% never consumed any type of alcoholic beverage, 30% stopped consuming it and 10% still consumed it socially; 93% consumed fruits, vegetables and legumes; and 77% did not practice any type of physical activity. Muller (2015) showed that, of the 407 individuals post-stroke living in Joinville-SC included in the study, 22% were smokers and 48% had never smoked. Furthermore, Lopes et al. (2022) showed that, of the 50 individuals post-stroke living in Belo Horizonte-MG included in the study that is still ongoing, 86% did not smoke, 76% did not consume alcoholic beverages, 68% stated that they had adequate diet, 62% consumed 2-3 fruits/vegetables/day; and only 36% practiced physical activity. As can be seen in these Brazilian studies, regular physical activity is the healthy lifestyle behavior least adopted by individuals post-stroke.

Several benefits related to the management of risk factors are associated with the regular practice of physical activity by individuals post-stroke, such as, for example, the reduction of mortality, blood pressure, cholesterol levels and blood glucose levels (Billinger et al., 2014, Prior et al., 2017, D'Isabella et al., 2017, Loprinzi, 2015, Saunders et al., 2014). Furthermore, recently, Lennon et al. (2021) investigated the predictors of adherence to lifestyle recommendations for stroke secondary prevention and identified that the variable cardiovascular fitness (measure operationalized as peak oxygen consumption, $VO2_{peak}$) was the only significant predictor of adherence to three health behaviors: healthy diet, physical activity and smoking cessation. Improved cardiovascular fitness is achieved through regular physical activity (Billinger et al., 2014). Therefore, these findings reinforce the importance of encouraging regular physical activity by individuals post-stroke when aiming to also improve adherence to other healthy behaviors. However, despite these benefits already described and considering the data presented previously, it has been identified that individuals post-stroke do not adopt the behavior of regular physical activity (Billinger et al., 2014).

Several barriers have been identified regarding individuals post-stroke not engaging in regular physical activity (Billinger et al., 2014). Some examples include: lack of interest or motivation, and lack of perceived self-confidence and self-efficacy (Billinger et al., 2014). Furthermore, a lack of knowledge about how and where to exercise and the potential benefits of physical activity has also been identified (Billinger et al., 2014). These barriers point to the need for actions that are contextualized and individualized, considering the capabilities, needs and priorities of individuals. A fact that has already been guided in the literature (Lawrence et al., 2019). To overcome these

barriers, it is important that theoretically-informed strategies are implemented (Lawrence et al., 2019).

Studies on strategies for changing the behavior of individuals post-stroke to adopt healthy behaviors, including regular physical activity, have currently been carried out (Bridgwood et al., 2018, Heron et al., 2017, Katsanos et al., 2017, Lennon et al., 2014, Lager et al., 2014). However, it is important to consider that behavior change interventions are typically complex and involve several interactive components (Craig et al., 2008). As a result, these interventions can be difficult to implement and replicate both in research and clinical practice (Michie et al., 2015). Therefore, there is a need to implement theoretically-informed strategies, aiming for a greater possibility of achieving the expected results, and adequate replication of interventions both in research and clinical practice (Michie et al., 2015). In this sense, Michie et al. (2015) developed a taxonomy for reporting and describing behavior change interventions. However, the use of theoretically-informed strategies for stroke secondary prevention aimed at adopting healthy lifestyle behaviors is not evident.

A recently published scoping review examined national guidelines for the care of individuals post-stroke and audit documents across all World Health Organization (WHO) regions to identify recommendations for non-surgical and non-pharmacological interventions for stroke secondary prevention and associated performance indicators (Hall et al., 2022). The authors found that these interventions for stroke secondary prevention are not a current pillar in national guidelines for the care of individuals post-stroke and audit documents (Hall et al., 2022). This scenario is even more serious considering underdeveloped and developing countries, such as Brazil, where fewer guidelines and documents were identified (Hall et al., 2022). Therefore, despite recent recommendations and guidelines for implementing theoretically-informed behavior change techniques for stroke secondary prevention, this has possibly not been applied in research and clinical practice worldwide. Furthermore, this scoping review showed that, of the 16 guidelines and documents identified, 11 recommended the practice of physical activity (Hall et al., 2022). However, the guidelines are still shallow, and no guidelines for treatment or stroke secondary prevention were found that provided specific guidelines for prescribing physical activity in Brazil (Hall et al., 2022). Therefore, in the Brazilian context, individuals post-stroke should still receive general guidelines for regular physical activity, which may not be effective in resulting in an increase in the level of physical activity in this population.

The Pan American Health Organization (PAHO) recently published a technical package called HEARTS for the management of cardiovascular diseases, including a specific document containing guidelines for adopting healthy behaviors, translated into Brazilian Portuguese (OPAS, 2019). This technical package consists of six modules that correspond to each of the letters of the HEARTS acronym (OPAS, 2019). Module H, “Healthy-lifestyle”, which includes patient counseling (OPAS, 2019). Module E, “Evidence”, which comprises the use of evidence-based protocols (OPAS, 2019). Module A, “Access”, which comprises access to essential medicines and technologies (OPAS, 2019). The R module, “Risk”, which comprises risk-based management of cardiovascular diseases (OPAS, 2019). The “T” module, which comprises team “Team” as the basis for care (OPAS, 2019). And, finally, module “S”, “Systems for monitoring” (OPAS, 2019).

In this technical package, the four main behavioral risk factors for cardiovascular diseases are described: unhealthy diet, smoking, harmful alcohol consumption and insufficient physical activity (OPAS, 2019). For healthy levels of physical activity for adults, the HEARTS technical package highlights the guidance that all individuals, including individuals post-stroke, perform at least: 150 minutes of moderate physical activity or at least 75 minutes of vigorous physical activity, distributed throughout the week; or an equivalent combination of moderate and vigorous activity; or muscle strengthening activities, exercising large muscle groups on two or more days of the week (OPAS, 2019).

The HEARTS technical package presents aspects related to behavior change and presents the brief intervention model using the 5As tool: ask, advise, assess, assist and arrange (OPAS, 2019). The use of this tool is recommended to encourage behavioral changes (OPAS, 2019). Considering the taxonomy for behavior change techniques proposed by Michie et al. (2013), the HEARTS technical package addresses those called goals and planning, feedback and monitoring, and social support (OPAS, 2019). To date, no study has been identified that used this HEARTS technical package to increase the level of physical activity in individuals post-stroke

In Brazil, only one clinical trial was identified in Clinical Trials with the objective of promoting physical activity in individuals post-stroke (Scianni, 2022). This clinical trial is being carried out with individuals post-stroke in the acute and subacute phase using theoretically-informed strategies (Scianni, 2022). However, guidelines recommend that physical activity should be a component of the ongoing care of individuals post-stroke (Billinger et al., 2014). In this sense, it is also necessary to promote the practice of

physical activity with individuals post-stroke in the chronic phase, using theoretically-informed strategies. Furthermore, no study was found that used the HEARTS technical package with the aim of increasing the level of physical activity in individuals post-stroke. Considering that this HEARTS technical package was translated into Brazilian Portuguese, its use can contribute to achieving significant results. Finally, the literature has also been scarce regarding the promotion of physical activity with individuals post-stroke in the chronic phase using a combination of strategies, such as the use of pedometers associated with another strategy (Lynch et al., 2018).

In addition to identifying the need to encourage the practice of physical activity by individuals post-stroke, it is important to ensure that this practice is continuous (Billinger et al., 2014). A possible strategy that can contribute to solving this problem is the use of telehealth. Telehealth refers to the provision of rehabilitation services in a remote location using information and communication technologies (Brennan et al., 2009). The use of telehealth interventions has been adopted in the health care of individuals post-stroke and has become more frequent with the COVID-19 pandemic (Bloem et al., 2020). Theoretically-informed interventions for self-management of lifestyle-related risk factors with individuals post-stroke, such as to increase physical activity level (Lawrence et al., 2019), can be delivered remotely via telehealth (Gladstone et al., 2022). However, recommendations for the use of telehealth interventions for behavior change and self-management in stroke secondary prevention are based on evidence classified as level C (Gladstone et al., 2022). Therefore, it is necessary to investigate the effect of interventions to increase the level of physical activity of individuals post-stroke via telehealth to better recommend the literature regarding the use of this intervention.

1.1 OBJECTIVES

Primary objective: To investigate whether a theoretically-informed telehealth intervention involving the HEARTS technical package and the use of an activity monitor to increase physical activity level is feasible to implement with individuals post-stroke.

Secondary objective: Estimate the parameters for carrying out a randomized clinical trial (RCT) considering the outcomes level of physical activity and number of individuals post-stroke who became physically active, systolic blood pressure (SBP) and diastolic blood pressure (DBP), cardiorespiratory fitness, self-efficacy for physical activity and health-related quality of life.

1.2 Study hypotheses

H0: A theoretically-informed telehealth intervention involving the HEARTS technical package and the use of an activity monitor to increase the level of physical activity is not feasible to implement with individuals post-stroke.

H1: A theoretically-informed telehealth intervention involving the HEARTS technical package and the use of an activity monitor to increase the level of physical activity is feasible to implement with individuals post-stroke.

2. METHODOLOGY

2.1 Design

This is a phase 1 feasibility study to conduct an RCT (Eldridge et al., 2016), with a blinded evaluator and concealed allocation. The project will be submitted to the Research Ethics Committee of the Universidade Federal de Minas Gerais (UFMG). The cover page is attached (ANNEX 1). All necessary consents for the development of the study were obtained (ANNEX 2). Furthermore, this project was approved by the UFMG Physiotherapy Department chamber (ANNEX 3).

This study will be registered at www.ClinicalTrials.gov and carried out in accordance with CONSORT extension recommendations for pilot or feasibility studies (Eldridge et al., 2016). Recruitment of individuals and data collection will begin after approval by the Research Ethics Committee of the UFMG.

All individuals will be instructed on the procedures that will be carried out and will only be included after agreeing to voluntary participation and signing the Informed Consent Form (ICF) (APPENDIX 1).

2.2 Participants

A convenience sample will be recruited in the community of the city of Belo Horizonte, through contact with outpatient clinics, clinics, hospitals, health centers, associations, research groups and extension projects developed with the population of interest.

To participate in the study, individuals must meet the following inclusion criteria:

- Present a clinical diagnosis of stroke more than six months ago;
- Be 18 years old or over;
- Be classified as “Inactive” according to the Adjusted Activity Score (AAS) of the Human Activity Profile (HAP) (Souza et al., 2006);

- Be able to walk 10 meters independently with or without a walking aid (considering that walking is a potential way to participate in physical activity);
- Present medical clearance to practice physical activity.

The following individuals will be excluded:

- Those who test positive for possible cognitive impairment (Mini-Mental State Examination) using the cutoff point based on educational level (Bertolucci et al., 1994) and/or have comprehension aphasia, assessed by the ability to respond to verbal commands using body movements (“raise your unaffected arm and open your hand”) (Teixeira-Salmela et al., 2007);
- Those with the presence of pain or other adverse health conditions that compromise the implementation of the proposed intervention program, such as vestibular disorders, severe arthritis, or any other diagnosed nervous system disease.

2.3 Randomization and concealed allocation

Participants will be allocated into two groups (experimental and control). The allocation sequence will be generated by a website (www.randomization.com). A research assistant, who will not be involved in recruitment, will perform the randomization and place the sequence in opaque, numbered, sealed envelopes. Envelopes will be prepared prior to the start of the study by a research assistant not involved in the research. Each participant will be allocated to one of the groups respecting the contents inside the envelopes. To minimize possible bias resulting from participants not being blinded, they will be instructed not to comment to the examiner about the group to which they were assigned.

2.4 Intervention

Participants will undergo an intervention program involving the HEARTS technical package (OPAS, 2019) and the experimental group will have the addition of self-monitored physical activity using the Smartwatch Mi Band 7®. Therefore, the only difference between the groups is that participants in the control group will not receive the physical activity monitor (Smartwatch Mi Band 7®). To monitor adherence to the protocol, individuals will receive a diary of regular physical activity, which will be filled

out, for each day of physical activity, the duration and the subjective effort score according to the Modified Borg Categorical Scale (Borg, 1982, Cavallazzi et al., 2008) (APPENDIX 2). The researcher responsible for implementing the intervention (physiotherapist with previous experience in caring for individuals post-stroke and one of the researchers responsible for the present study) and a research assistant (scientific initiation students from the undergraduate physiotherapy course) will be trained in advance for standardization. implementation of intervention protocols, which will always be carried out by the same therapist. A companion/caregiver will be instructed to assist the individual in filling out the diary, if necessary.

2.4.1 Experimental group

Participants in the experimental group will carry out an intervention program involving the HEARTS technical package (OPAS, 2019) and the practice of self-monitored physical activity. The HEARTS technical package presents the model for implementing the 5As brief intervention to increase physical activity (OPAS, 2019). This method involves: ask, advise, assess, assist and arrange (OPAS, 2019). Participants in the experimental group will receive a physical activity monitor (Smartwatch Mi Band 7®) (as instructed in the "assist" topic in the HEARTS technical package) and will be monitored by telephone, once a week, aiming to identify successes and difficulties, as well as, to reinforce successes and identify solutions to identified difficulties (as guided in the "arrange" topic in the HEARTS technical package) (PAHO, 2019). These individuals will undertake a 12-week intervention program. In the first week, individuals will carry out the first four stages of the 5As brief intervention in a meeting that will be held in person at the participant's home or at the Laboratório de Estudos em Reabilitação Neurológica do Adulto (NEUROLAB) at Escola de Educação Física, Fisioterapia, e Terapia Ocupacional (EEFFTO) (UFMG), depending on the participant's availability, where they will be instructed on how to use the physical activity monitor (they will be instructed to use it during the time they are awake, every day, during the study), they will receive the diary of regular physical activity practice (presented in the previously developed evaluation form, APPENDIX 2), will be guided on how to fill it out, and will schedule the best times to receive the weekly telephone call.

2.4.2 Control group

Participants in the control group will perform the same intervention as the experimental group, but will not receive the physical activity monitor (Smartwatch Mi Band 7®).

2.5 Measurement of outcomes

The measurement of primary and secondary outcomes will be carried out in NEUROLAB. Measurements will be carried out at the initial assessment (week 0), immediately after the end of the intervention program (week 12) and one month after the end of the intervention program (week 16). In the initial assessment (week 0), clinical and demographic data will be collected from all individuals for the purpose of verifying the eligibility criteria, identification and characterization of the sample. For this, a previously developed evaluation form will be used (APPENDIX 2). In weeks 12 and 16, a previously developed reassessment form similar to the evaluation form will be used, in addition to the addition of an assessment of the participant's satisfaction with the intervention carried out (APPENDIX 3). This collection will be carried out by a previously trained examiner, blinded to group allocation. All participants will be instructed not to comment on information about the training received.

2.5.1 Primary outcomes: feasibility measures

Recruitment feasibility

The feasibility of recruitment will be determined by the ratio between the total number of eligible individuals and the total number of individuals screened, and by the ratio between the total number of eligible individuals and the total number of individuals recruited (Caetano et al., 2023, Peniche et al., 2022, Kim et al., 2020, Regan et al., 2021).

Feasibility of the intervention

The feasibility of the intervention will be determined by examining retention, follow-up of individuals, attendance, safety, and perceived effectiveness.

Retention will be determined by the ratio of the total number of individuals who completed the proposed intervention program to the total number of individuals who began the proposed intervention program. The reasons for dropping out of the intervention program will be recorded (Peniche et al., 2022, Kim et al., 2020, Regan et al., 2021, Hanlon et al., 2022).

The follow-up of individuals will be determined by the ratio between the total number of individuals who remained in the same group to which they were initially allocated until follow-up and the total number of individuals who were allocated (Regan et al., 2021).

Attendance will be determined by the ratio between the total number of sessions held and the total number of sessions offered. The number and reasons for absence from sessions will be recorded (Caetano et al., 2023, Peniche et al., 2022, Regan et al., 2021).

Safety will be determined by the number and reasons for adverse events (e.g., pain, falls, hospitalization, and death) identified during the individual's intervention and follow-up period (Caetano et al., 2023, Regan et al., 2021, Hanlon et al., 2022).

Perceived effectiveness will be determined by the following question: “Comparing how it was before you performed the intervention and now, do you think your ability to perform routine physical activity is: much worse, moderately worse, a little worse, the same, a little better, moderately better or much better?” The answer provided will be recorded by the independent examiner.

Feasibility of obtaining clinical measurements

The feasibility of obtaining clinical measurements will be determined by the percentage of clinical outcomes measured (ratio between the number of clinical outcomes measured and the number of clinical outcomes proposed to be measured) and the percentage of participants who completed the diary (ratio between the number of individuals who completed the diary and the number of individuals who started the proposed intervention program) (Caetano et al., 2023).

2.5.2 Secondary outcomes: clinical measures

Clinical outcomes will be evaluated to estimate the parameters for carrying out an RCT, including: level of physical activity and number of individuals post-stroke who became physically active, SBP and DBP, cardiorespiratory fitness, self-efficacy for physical activity and health-related quality of life.

The level of physical activity and the number of individuals post-stroke who became physically active will be measured with the HAP (Souza et al., 2006). The HAP is a questionnaire, applied in the form of an interview, consisting of 94 activities, each one graded hierarchically according to the metabolic equivalent required (Teixeira-Salmela et al., 2007, Souza et al., 2006). Activities include personal care, transportation,

home maintenance, social and leisure activities, and physical exercise. For each item, there are three possible answers: “still does the activity”, “stopped doing it” and “never did it” (Teixeira-Salmela et al., 2007, Souza et al., 2006). The maximum activity score (MAS) indicates the activity with the highest energy expenditure that the individual is capable of performing (Teixeira-Salmela et al., 2007, Souza et al., 2006). An AAS is obtained by subtracting from the maximum score the number of activities that the individual stopped performing and indicates the average level of typical metabolic equivalent (Teixeira-Salmela et al., 2007, Souza et al., 2006). Higher scores indicate better results (Souza et al., 2006). The level of physical activity will be operationalized by the AAS, in points (Souza et al., 2006). Another measure of the HAP is the “Activity Classification”, which provides an overall classification of the individual's activity level into “Inactive”, “Moderately active” and “Active”. The number of individuals who have become “Moderately Active” and “Active” will be identified. The HAP is an instrument already adapted to Portuguese-Brazil, which presents appropriate measurement properties for assessing the level of physical activity of individuals post-stroke (Teixeira-Salmela et al., 2007, Souza et al., 2006).

SBP and DBP will be operationalized as the average of the pressures obtained. These measurements will be obtained using the Tycos® aneroid sphygmomanometer (WelchAllyn Inc., NY, USA, Model DS-44) and the stethoscope (Litmann Classic II SE 3M®, USA).

Cardiorespiratory fitness will be measured by HAP (Souza et al., 2006). One of the HAP measures is “Lifestyle Energy Consumption”, operationalized in $ml.kg^{-1}.min^{-1}$ (Souza et al., 2006). The HAP is a valid instrument for estimating the cardiorespiratory fitness of individuals post-stroke (Brito et al., 2022).

Self-efficacy for physical activity will be operationalized considering the score obtained on the Self-Efficacy for Physical Activity Scale (Rech et al., 2011). No instrument was found that presents adequate measurement properties to assess self-efficacy for physical activity in individuals post-stroke. The scale presents 10 questions, divided into two sections (Rech et al., 2011). Each question must be answered as yes (1 point) or no (0 point) (Rech et al., 2011). Scores are computed with the sum of the answers to each question (Rech et al., 2011). The higher the value, the higher the self-efficacy for practicing physical activity (Rech et al., 2011). Rech et al. (2011) investigated the measurement properties of the scale and identified that it presents adequate validity,

internal consistency and reproducibility to assess self-efficacy for physical activity in Brazilian adults.

Health-related quality of life will be operationalized considering the scores obtained on the Stroke-Specific Quality of Life Scale (SS-QOL) (Ciconelli et al., 1999, Lima et al., 2008). The Brazilian version of the SS-QOL has twelve domains (energy, family role, language, mobility, humor, personality, self-care, social role, reasoning, upper limb function, vision and work/productivity), totaling 49 items (Lima et al ., 2008). There are three possible answers, on a scale of five to one: amount of help needed to perform specific tasks, amount of difficulty experienced when it is necessary to perform a task and degree of agreement with statements about functionality (Lima et al., 2008). The minimum possible score is 49 (worst perception of health-related quality of life) and the maximum score is 245 (best perception of health-related quality of life) (Lima et al., 2008). The reference for responses is the previous week (Lima et al., 2008). This instrument is applied in the form of an interview, can be administered in a short time and has good clinical applicability and adequate measurement properties for evaluating this outcome in individuals post-stroke (Ciconelli et al., 1999, Lima et al., 2008, Cabral et al., 2012). This instrument has already been translated and adapted into Brazilian Portuguese (Ciconelli et al., 1999, Lima et al., 2008).

2.6 Sample calculation

As a feasibility study, no formal sample size calculation is performed (Billingham et al., 2013). Following the recommendation proposed by Julious (2005) for feasibility studies with parallel groups, the present study will present a sample size of 24 individuals, with 12 individuals in each group.

2.7 Procedures

During the execution of the study, measures to prevent the spread of the coronavirus will be observed (COMITÊ PERMANENTE DE ENFRENTAMENTO DO NOVO CORONAVÍRUS DA UFMG, 2020; MINISTÉRIO DA SAÚDE, 2020). Individuals will be scheduled by time to avoid crowds. When scheduling assessments, a screening will be carried out to assess the presence of flu-like symptoms. In the presence of symptoms, the isolation period will be respected, and the individual will be contacted again after the safety period. Evaluators will use disposable surgical masks. Participants will also be asked to wear masks, and if necessary, procedural masks will be provided for

use during the assessment. When necessary, the mask can be removed to carry out the tests. All reusable equipment will be properly sanitized and sterilized. Finally, 70% alcohol gel will be made available for hand hygiene (COMITÊ PERMANENTE DE ENFRENTAMENTO DO NOVO CORONAVÍRUS DA UFMG, 2020; MINISTÉRIO DA SAÚDE, 2020).

Individuals interested in voluntarily participating in this study will be assessed for eligibility according to the criteria described previously. Those who meet the established criteria will be randomly allocated to an experimental group or control group. All data will be collected by the same previously trained evaluator, with the help of a research assistant, who will be blinded regarding the allocation of participants. Another researcher with the assistance of a research assistant will be responsible for administering the intervention and will be blinded to participant data.

This study will be carried out at the NEUROLAB of the Department of Physiotherapy of the EEFFTO (UFMG).

2.8 Statistical Analysis

All statistical analyzes will be carried out by an independent examiner, blinded to all procedures carried out in the study, including group allocation. Descriptive statistics will be calculated for all results. Effect sizes will be calculated to determine the magnitude of within- and between-group comparisons. All analyzes will be carried out using the SPSS 20.0 statistical program and α=0.05 will be considered, adjusted for multiple comparisons.

REFERENCES

Bertolucci PH, Brucki SM, Campacci SR, et al. O Mini-Exame do Estado Mental em uma população geral. Impacto da escolaridade. Arq Neuropsiquiatr. 1994;52(1):1-7.

Billinger S, Arena R, Bernhardt J, et al. Physical activity and exercise recommendations for stroke survivors: a statement for healthcare professionals from the American Heart Association/American Stroke Association. Stroke. 2014;45(8):2532-53.

Billingham SA, Whitehead AL, Julious SA. An audit of sample sizes for pilot and feasibility trials being undertaken in the United Kingdom registered in the United Kingdom Clinical Research Network database. BMC Med Res Methodol. 2013;13:104.

Bloem BR, Dorsey ER, Okun MS. The Coronavirus Disease 2019 Crisis as Catalyst for Telemedicine for Chronic Neurological Disorders. JAMA Neurol. 2020;77(8):927-28.

Borg GA. Psychophysical bases of perceived exertion. Med Sci Sports. 1982:14(5):377-81.

Brennan DM, Mawson S, Brownsell S. Telerehabilitation: enabling the remote delivery of healthcare, rehabilitation, and self management. Stud Health Technol Inform. 2009;145:231-48.

Bridgwood B, Lager KE, Mistri AK, et al. Interventions for improving modifiable risk factor control in the secondary prevention of stroke. Cochrane Database Syst Rev. 2018;5(5):CD009103.

Brito SAF, Aguiar LT, Quintino LF, et al. Assessment of $\dot{V}$o(2peak) and Exercise Capacity After Stroke: A Validity Study of the Human Activity Profile Questionnaire. Arch Phys Med Rehabil. 2022;103(9):1771-6.

Cabral DL, Laurentino GE, Damascena CG, et al. Comparisons of the Nottingham Health Profile and the SF-36 health survey for the assessment of quality of life in individuals with chronic stroke. Rev Bras Fisioter. 2012;16(4):301-8.

Cadilhac DA, Prvu Bettger J. Health Policy and Health Services Delivery in the Era of COVID-19. Stroke. 2021;52(6):2177-79.

Caetano LC, Ada L, Romeu Vale S, et al. Self-management to promote physical activity after discharge from in-patient stroke rehabilitation: a feasibility study. Top Stroke Rehabil. 2023;30(1):32-42.

Carvalho-Pinto BP, Faria CD. Health, function and disability in stroke patients in the community. Braz J Phys Ther. 2016;20(4):355-66.

Cavallazzi TG L, Cavallazzi RS, Cavalcante YMC, et al. Avaliação do uso da Escala Modificada de Borg na crise asmática. Acta Paul Enferm. 2005;18(1):39-45.

Ciconelli RM, Ferraz MB, Santos W, et al. Tradução para a língua portuguesa e validação do questionário genérico de qualidade de vida SF-36 (Brasil SF-36). Rev Bras Reumatol. 1999;39(3):143-50.

COMITÊ PERMANENTE DE ENFRENTAMENTO DO NOVO CORONAVÍRUS DA UFMG, 2020. Protocolo de biossegurança e adequação do espaço físico na UFMG. 2020.

Craig P, Dieppe P, Macintyre S, et al. Developing and evaluating complex interventions: the new Medical Research Council guidance. BMJ 2008;337:a1655.

D'Isabella NT, Shkredova DA, Richardson JA, et al. Effects of exercise on cardiovascular risk factors following stroke or transient ischemic attack: a systematic review and meta-analysis. Clin Rehabil. 2017;31(12):1561–72

Damsbo AG, Kraglund KL, Buttenschøn HN, et al. Predictors for wellbeing and characteristics of mental health after stroke. J Affect Disord. 2020;264:358-64.

Eldridge SM, Chan CL, Campbell MJ, et al. CONSORT 2010 statement: extension to randomised pilot and feasibility trials. Bmj. 2016;355:i5239.

Eldridge SM, Lancaster GA, Campbell MJ, et al. Defining Feasibility and Pilot Studies in Preparation for Randomised Controlled Trials: Development of a Conceptual Framework. PLoS One. 2016;11(3):e0150205.

Feigin VL, Brainin M, Norrving B, et al. World Stroke Organization (WSO): Global Stroke Fact Sheet 2022. Int J Stroke. 2022;17(1):18-29.

Feigin VL, Stark BA, Johnson CO, et al. Global, regional, and national burden of stroke

and its risk factors, 1990-2019: a systematic analysis for the Global Burden of Disease Study 2019. Lancet Neurol. 2021;20(10):795-820.

Gittler M, Davis AM. Guidelines for Adult Stroke Rehabilitation and Recovery. JAMA. 2018;319(8):820-21.

Gladstone DJ, Lindsay MP, Douketis J, et al. Canadian Stroke Best Practice Recommendations: Secondary Prevention of Stroke Update 2020. Can J Neurol Sci. 2021:1-23.

Hall P, von Koch L, Wang X, et al. A Scoping Review of Non-Pharmacological, Non-Surgical Secondary Prevention Strategies in Ischaemic Stroke and TIA in National Stroke Guidelines and Clinical Audit Documents. Healthcare (Basel). 2022;10(3):481.

Hanlon SL, Bley BC, Silbernagel KG. Determining the feasibility of exercise therapy and activity modification for treating adolescents with heel pain: a study protocol. BMJ Open Sport Exerc Med. 2022;8(3):e001301.

Heron N, Kee F, Cardwell C, et al. Secondary prevention lifestyle interventions initiated within 90 days after TIA or 'minor' stroke: a systematic review and meta-analysis of rehabilitation programmes. Br J Gen Pract. 2017;67(654):e57-e66.

Julious SA. Sample size of 12 per group rule of thumb for a pilot study. Pharmaceut Statist. 2005;4(4):287–91.

Katsanos AH, Filippatou A, Manios E, et al. Blood Pressure Reduction and Secondary Stroke Prevention: A Systematic Review and Metaregression Analysis of Randomized Clinical Trials. Hypertension. 2017;69(1):171-79.

Kim BJ, Park JM, Park TH, et al. Remote blood pressure monitoring and behavioral intensification for stroke: A randomized controlled feasibility trial. PLoS One. 2020;15(3):e0229483.

Kleindorfer DO, Towfighi A, Chaturvedi S, et al. 2021 Guideline for the Prevention of Stroke in Patients With Stroke and Transient Ischemic Attack: A Guideline From the American Heart Association/American Stroke Association. Stroke. 2021;52(7):e364-e467.

Lager KE, Mistri AK, Khunti K, et al. Interventions for improving modifiable risk factor control in the secondary prevention of stroke. Cochrane Database Syst Rev. 2014;(5):CD009103.

Lawrence M, Asaba E, Duncan E, et al. Stroke secondary prevention, a non-surgical and non-pharmacological consensus definition: results of a Delphi study. BMC Res Notes. 2019;12(1):823.

Lawrence M, Pringle J, Kerr S, et al. Multimodal secondary prevention behavioral interventions for TIA and stroke: a systematic review and meta-analysis. PLoS One. 2015;10(3):e0120902.

Lennon O, Galvin R, Smith K, et al. Lifestyle interventions for secondary disease prevention in stroke and transient ischaemic attack: a systematic review. Eur J Prev Cardiol. 2014;21(8):1026-39.

Lennon O, Hall P, Blake C. Predictors of Adherence to Lifestyle Recommendations in Stroke Secondary Prevention. Int J Environ Res Public Health. 2021;18(9):4666.

Lima R, Teixeira-Salmela LF, Magalhaes L, et al. Propriedades da versão brasileira do stroke specific quality of life: aplicação do modelo Rasch. Rev Bras Fisioter. 2008;12(2):149-56.

Lin B, Zhang Z, Mei Y, et al. Cumulative risk of stroke recurrence over the last 10 years: a systematic review and meta-analysis. Neurol Sci. 2021;42(1):61-71.

Lopes MEM, Santos JM, Lima LAO, et al. PERFIL DE HÁBITOS SAUDÁVEIS DE INDIVÍDUOS PÓS ACIDENTE VASCULAR CEREBRAL E SEU CONHECIMENTO SOBRE ESTA CONDIÇÃO DE SAÚDE: RESULTADOS PRELIMINARES. Resumo: XXXI Semana de Iniciação Científica. Belo Horizonte: Universidade Federal de Minas Gerais, 2022.

Loprinzi P. Dose-response association of moderate-to-vigorous physical activity with cardiovascular biomarkers and all-cause mortality: considerations by individual sports, exercise and recreational physical activities. Prev Med. 2015;81:73–7.

Lynch EA, Jones TM, Simpson DB, et al. Activity monitors for increasing physical

activity in adult stroke survivors. Cochrane Database Syst Rev. 2018;7(7):CD012543.

Michie S, Richardson M, Johnston M, et al. The behavior change technique taxonomy (v1) of 93 hierarchically clustered techniques: building an international consensus for the reporting of behavior change interventions. Ann Behav Med. 2013;46(1):81-95.

Michie S, Wood CE, Johnston M, et al. Behaviour change techniques: the development and evaluation of a taxonomic method for reporting and describing behaviour change interventions (a suite of five studies involving consensus methods, randomised controlled trials and analysis of qualitative data). Southampton (UK): NIHR Journals Library, 2015.

Modrego PJ, Pina MA, Fraj MM, et al. Type, causes, and prognosis of stroke recurrence in the province of Teruel, Spain. A 5-year analysis. Neurol Sci. 2000;21(6):355-60.

Muller M. RECORRÊNCIA E LETALIDADE DO ACIDENTE VASCULAR CEREBRAL EM JOINVILLE, BRASIL: ESTUDO PROSPECTIVO DE BASE POPULACIONAL. Dissertação de mestrado. Joinville: Universidade da Região de Joinville, 2015. Available from: http://univille.edu.br/account/ppgsma/VirtualDisk.html?action=readFile&file=Dissertacao_Milena_Muller.pdf&current=/Dissertacoes_completas/2015.

Organização Pan-Americana da Saúde (OPAS). HEARTS Pacote de medidas técnicas para manejo da doença cardiovascular na atenção primária à saúde. Guia de implementação. Washington: Organização Pan-Americana da Saúde, 2019.

Peniche PC, Pinto APS, Ribeiro RLMN, et al. Recruitment, retention, attendance, and adherence of a randomized controlled trial to evaluate the effects of task-specific training with individuals post stroke. Fisioter Pesqui. 2022;29(1):22-8.

Prior PL, Hachinski V, Chan R, et al. Comprehensive cardiac rehabilitation for secondary prevention after transient ischemic attack or mild stroke: psychological profile and outcomes. J Mol Signal. 2017;37(6):428–36.

Rech CR, Sarabia TT, Fermino RC, et al. Propriedades psicométricas de uma escala de autoeficácia para a prática de atividade física em adultos brasileiros. Rev Panam Salud Publica. 2011:29(4):259–66.

Regan EW, Handlery R, Stewart JC, et al. Feasibility of integrating survivors of stroke into cardiac rehabilitation: A mixed methods pilot study. PLoS One. 2021;16(3):e0247178.

Ribeiro KSQS, Neves RF, Brito GEG, et al. PERFIL DE USUÁRIOS ACOMETIDOS POR ACIDENTE VASCULAR CEREBRAL ADSCRITOS À ESTRATÉGIA SAÚDE DA FAMÍLIA EM UMA CAPITAL DO NORDESTE DO BRASIL. RBCS. 2013;16:35-44.

Saunders D, Greig C, Mead G. Physical activity and exercise after stroke: Review of multiple meaningful benefits. Stroke. 2014;45:3742–47.

Scianni AA. Promoting Physical Activity After Stroke Via Self-management. ClinicalTrials.gov. 2022. Available from: https://clinicaltrials.gov/ct2/show/study/NCT05461976?cond=Stroke&cntry=BR&draw=2&rank=1.

Souza AC, Magalhães LC, Teixeira-Salmela LF. Adaptação transcultural e análise das propriedades psicométricas da versão brasileira do Perfil de Atividade Humana. Cad Saude Publica. 2006;22(12):2623-36.

Stroke Association, James Lind Alliance. Priorities in stroke rehabilitation and long-term care. 2021. Available from: https://www.stroke.org.uk/sites/default/files/research/priorities_in_stroke_rehabilitation_and_lon-term_care.pdf.

Teixeira-Salmela LF, Devaraj R, Olney SJ. Validation of the human activity profile in stroke: a comparison of observed, proxy and self-reported scores. Disabil Rehabil. 2007;29(19):1518-24.

Trabaquini D. CONSUMO ALIMENTAR DE PESSOAS APÓS SOFREREM ACIDENTE VASCULAR CEREBRAL. Trabalho de conclusão do curso (Enfermagem). Assis: Fundação Educacional do Município de Assis-FEMA, 2015. Available from: https://cepein.femanet.com.br/BDigital/arqTccs/1111370129.pdf.

Wang A, Wu L, Wang X, et al. Effect of recurrent stroke on poor functional outcome in transient ischemic attack or minor stroke. Int J Stroke. 2016;11(7):NP80.

Winstein CJ, Stein J, Arena R, et al. Guidelines for Adult Stroke Rehabilitation and Recovery: A Guideline for Healthcare Professionals From the American Heart Association/American Stroke Association. Stroke. 2016;47(6):e98-e169.

Zhong W, Geng N, Wang P, et al. Prevalence, causes and risk factors of hospital readmissions after acute stroke and transient ischemic attack: a systematic review and meta-analysis. Neurol Sci. 2016;37(8):1195-202.
