# Supplementary material for: Telehealth intervention involving the HEARTS Technical Package and the additional use of an activity monitor to increase physical activity level post-stroke: Protocol for a feasibility randomized controlled trial
Source: PLoS One. 2025 Apr 4;20(4):e0320026. doi: 10.1371/journal.pone.0320026 (PMC11970671; doi:10.1371/journal.pone.0320026)
Supplement: S2 File — (PDF) [file pone.0320026.s002.pdf]

IMPLEMENTAÇÃO DE UMA INTERVENÇÃO TEORICAMENTE INFORMADA POR TELESSAÚDE PARA AUMENTAR O NÍVEL DE ATIVIDADE FÍSICA DE INDIVÍDUOS PÓS-ACIDENTE VASCULAR ENCEFÁLICO: VIABILIDADE DE UM ENSAIO CLÍNICO ALEATORIZADO

Pesquisadora coordenadora: Profª Christina Danielli Coelho de Morais Faria, PhD, docente em atividade na Universidade Federal de Minas Gerais (UFMG), com vínculo permanente, na unidade Escola de Educação Física, Fisioterapia e Terapia Ocupacional, Departamento de Fisioterapia.

Área de conhecimento: Ciências da Saúde

Belo Horizonte

Universidade Federal de Minas Gerais

Escola de Educação Física, Fisioterapia e Terapia Ocupacional

Departamento de Fisioterapia

2023

1. INTRODUÇÃO

O acidente vascular encefálico (AVE) é uma condição de saúde que apresenta elevada carga em todo o mundo, principalmente em países subdesenvolvidos e em desenvolvimento, como o Brasil (Feigin *et al.*, 2021). Essa elevada carga está associada à alta incidência e prevalência que esta condição de saúde apresenta e às importantes incapacidades que comprometem a saúde e o bem-estar dos indivíduos (Feigin *et al.*, 2021, Damsbo *et al.*, 2020, Carvalho-Pinto *et al.*, 2016). Mais de 12 milhões de novos casos de AVE ocorrem anualmente, e tem sido estimado que uma a cada quatro pessoas com mais de vinte e cinco anos sofrerá um episódio de AVE durante a vida (Feigin *et al.*, 2022). Atualmente, existem mais de 100 milhões de pessoas sobreviventes ao AVE no mundo, sendo que 67% tem menos de 70 anos de idade (Feigin *et al.*, 2022). Além disso, tem sido identificado que, anualmente, mais de 143 milhões de ano de vida saudável são perdidos devido a mortes e incapacidades relacionadas ao AVE (Feigin *et al.*, 2022).

Outro fator contribuinte para que o AVE tenha elevada carga em todo o mundo é a sua recorrência (Feigin *et al.*, 2021, Lin *et al.*, 2021). O AVE recorrente apresenta elevada incidência e prevalência, é considerado a principal causa de readmissões hospitalares durante o primeiro ano após o episódio do AVE, resulta em um quadro de incapacidade ainda mais grave, e consequentemente, saúde, bem-estar e qualidade de vida mais prejudicados quando comparados a de indivíduos que sofreram um episódio de AVE, e contribui com um aumento de, aproximadamente, 40% dos custos em saúde (Lin *et al.*, 2021, Wang *et al.*, 2016, Zhong *et al.*, 2016, Modrego *et al.*, 2000). Estes fatos apontam para uma importante necessidade de saúde pública: ações para a prevenção secundária do AVE. Essa necessidade tem sido identificada pela *Stroke Association* e a *James Lind Alliance*, que apontaram a prevenção secundária do AVE como uma das 10 principais áreas de pesquisa prioritárias para reduzir a carga do AVE (*Stroke Association* e *James Lind Alliance*, 2021). Além disso, essa prioridade tornou-se ainda mais evidente no contexto da pandemia da *Corona Virus Disease-19* (COVID-19), que comprometeu o processo de reabilitação de indivíduos pós-AVE (Cadilhac *et al.*, 2021).

Diretrizes orientam que o processo de reabilitação de indivíduos pós-AVE deve ter como objetivo mantê-los bem e livres de eventos adicionais (Gittler *et al.*, 2018, Winstein *et al.*, 2016). Essas diretrizes ressaltam, também, a importância de incluir intervenções direcionadas para a adoção de um estilo de vida saudável em programas de prevenção secundária do AVE (Gittler *et al.*, 2018, Winstein *et al.*, 2016). Diretrizes específicas sobre a prevenção secundária do AVE, além de orientarem a adoção de um

estilo de vida saudável, também recomendam a implementação de programas de intervenção multimodais (Gladstone *et al*., 2021, Kleindorfer *et al*., 2021). Programas de intervenção multimodais podem incluir, por exemplo, ações de educação em saúde relacionadas à adesão medicamentosa, ao conhecimento sobre o AVE e sobre fatores de risco para a ocorrência do AVE, e para o aumento da prática de atividade física (Lawrence *et al*., 2015). Porém, apesar das recentes orientações e recomendações, ainda não é frequente a implementação de ações para prevenção secundária do AVE.

Recentemente, o conceito de prevenção secundária do AVE baseado em intervenções não-cirúrgicas e não-farmacológicas foi definido utilizando o método *Delphi* (Lawrence *et al*., 2019). Essa definição aborda a melhora da saúde e bem-estar, bem como a redução do risco do AVE recorrente, baseada na implementação de intervenções e estratégias educacionais teoricamente informadas (Lawrence *et al*., 2019). Além disso, o conceito apresentado orienta que as intervenções direcionadas para o gerenciamento dos fatores de risco relacionados ao estilo de vida devem ser contextualizadas e individualizadas considerando as capacidades, necessidades e prioridades dos indivíduos, bem como de suas famílias (Lawrence *et al*., 2019). O gerenciamento do conjunto de fatores de risco relacionados ao estilo de vida, como tabagismo, consumo nocivo de bebida alcoólica, alimentação inadequada e inatividade física, tem sido considerado o pilar das estratégias de prevenção secundária do AVE (Gladstone *et al*., 2021, Kleindorfer *et al*., 2021, OPAS, 2020).

O gerenciamento desse conjunto de fatores de risco tem sido considerado o pilar das estratégias de prevenção secundária do AVE porque os fatores de risco comportamentais representam, aproximadamente, 47% da carga do AVE (Feigin *et al*., 2022). Além dos benefícios diretos alcançados pelo seu gerenciamento, benefícios indiretos relacionados ao gerenciamento de fatores de risco cardiovasculares também podem ser identificados (Gladstone *et al*., 2021, Kleindorfer *et al*., 2021, OPAS, 2020). Fatores de risco cardiovasculares incluem, por exemplo, hipertensão arterial, dislipidemia, hipercolesterolemia e diabetes (Feigin *et al*., 2022, Gladstone *et al*., 2021, Kleindorfer *et al*., 2021, OPAS, 2020). Porém, para melhor direcionamento da implementação de ações para prevenção secundária do AVE, é importante considerar, como apontado anteriormente, que essas ações devem ser contextualizadas e individualizadas considerando as capacidades, necessidades e prioridades dos indivíduos, bem como de suas famílias (Lawrence *et al*., 2019).

Ainda são poucos os estudos que detalham o contexto brasileiro quanto ao perfil

de adoção de hábitos de vida saudáveis relacionados ao pilar das estratégias de prevenção secundária do AVE: cessação do tabagismo, consumo seguro de bebida alcoólica, alimentação adequada e prática regular de atividade física. Ribeiro *et al.* (2012), em um dos poucos estudos desenvolvidos sobre esta temática no Brasil, mostraram que, dos 140 indivíduos pós-AVE residentes em João Pessoa-PB incluídos no estudo, 79% não eram etilistas e 84% não eram tabagistas. Trabaquini (2015) mostrou que, dos 30 indivíduos pós-AVE residentes em Andirá-PR incluídos no estudo, 70% nunca fumaram, 17% pararam de fumar e 13% eram fumantes; 60% nunca consumiram nenhum tipo de bebida alcóolica, 30% pararam de consumir e 10% ainda consumiam socialmente; 93% consumiam frutas, verduras e legumes; e 77% não realizavam nenhum tipo de atividade física. Muller (2015) mostrou que, dos 407 indivíduos pós-AVE residentes em Joinville-SC incluídos no estudo, 22% eram fumantes e 48% nunca fumaram. Além disso, Lopes *et al.* (2022) mostraram que, dos 50 indivíduos pós-AVE residentes em Belo Horizonte-MG incluídos no estudo que ainda está andamento, 86% não fumavam, 76% não consumiam bebida alcóolica, 68% afirmaram que tinham alimentação adequada, 62% consumiam 2-3 frutas/vegetais/dia; e apenas 36% praticavam atividade física. Como pode ser observado nestes estudos brasileiros, a prática regular de atividade física é o hábito de vida saudável menos adotado por indivíduos pós-AVE.

Diversos benefícios relacionados ao gerenciamento dos fatores de risco estão associados à prática regular de atividade física por indivíduos pós-AVE, como, por exemplo, a redução da mortalidade, da pressão arterial, dos níveis de colesterol e da glicemia (Billinger *et al.*, 2014, Prior *et al.*, 2017, D'Isabella *et al.*, 2017, Loprinzi, 2015, Saunders *et al.*, 2014). Além disso, recentemente, Lennon *et al.* (2021) investigaram os preditores da adesão às recomendações de estilo de vida para a prevenção secundária do AVE e identificaram que a variável aptidão cardiovascular (medida operacionalizada como consumo pico de oxigênio, VO_{2pico}) foi a única preditora significativa da adesão a três comportamentos de saúde: dieta saudável, atividade física e cessação do tabagismo. A melhora da aptidão cardiovascular é alcançada pela prática regular de atividade física (Billinger *et al.*, 2014). Portanto, esses achados reforçam a importância do incentivo à prática regular de atividade física por indivíduos pós-AVE quando se tem o objetivo de também melhorar a adesão aos demais hábitos saudáveis. Porém, apesar desses benefícios já descritos e considerando os dados apresentados anteriormente, tem-se identificado que indivíduos pós-AVE não adotam o hábito da prática regular de atividade física (Billinger *et al.*, 2014).

Diversas barreiras têm sido identificadas quanto a não realização da prática regular de atividade física por indivíduos pós-AVE (Billinger *et al.*, 2014). Alguns exemplos incluem: falta de interesse ou motivação, e falta de percepção de autoconfiança e autoeficácia (Billinger *et al.*, 2014). Além disso, a falta de conhecimento sobre como e onde se exercitar e sobre os potenciais benefícios da prática de atividade física também têm sido identificada (Billinger *et al.*, 2014). Essas barreiras apontam a necessidade de ações que sejam contextualizadas e individualizadas considerando as capacidades, necessidades e prioridades dos indivíduos. Fato que já tem sido orientado na literatura (Lawrence *et al.*, 2019). Para superar essas barreiras, é importante que estratégias teoricamente informadas sejam implementadas (Lawrence *et al.*, 2019).

Estudos sobre estratégias para mudança de comportamento de indivíduos pós-AVE para a adoção de hábitos saudáveis, dentre eles, a prática regular de atividade física, têm sido atualmente realizados (Bridgwood *et al.*, 2018, Heron *et al.*, 2017, Katsanos *et al.*, 2017, Lennon *et al.*, 2014, Lager *et al.*, 2014). Porém, é importante considerar que intervenções para mudança de comportamento são tipicamente complexas e envolvem diversos componentes interativos (Craig *et al.*, 2008). Decorrente disso, essas intervenções podem ser difíceis de serem implementadas e replicadas tanto na pesquisa quanto na prática clínica (Michie *et al.*, 2015). Por isso, há a necessidade de se implementar estratégias teoricamente informadas, visando maior possibilidade de atingir os resultados esperados, e adequada replicação das intervenções tanto na pesquisa quanto na prática clínica (Michie *et al.*, 2015). Nesse sentido, Michie *et al.* (2015) desenvolveram uma taxonomia para reportar e descrever intervenções para mudança de comportamento. Porém, ainda assim, não é evidente o uso de estratégias teoricamente informadas para prevenção secundária do AVE voltada para a adoção de hábitos de vida saudáveis.

Uma revisão de escopo recentemente publicada examinou diretrizes nacionais voltadas para o cuidado de indivíduos pós-AVE e documentos de auditoria em todas as regiões da Organização Mundial da Saúde (OMS) para identificar recomendações de intervenções não-cirúrgicas e não-farmacológicas para prevenção secundária do AVE e indicadores de desempenho associados (Hall *et al.*, 2022). Os autores encontraram que essas intervenções para prevenção secundária do AVE não são um pilar atual nas diretrizes nacionais voltadas para o cuidado de indivíduos pós-AVE e documentos de auditoria (Hall *et al.*, 2022). Esse cenário é ainda mais grave considerando países subdesenvolvidos e em desenvolvimento, como o Brasil, onde foi identificada menor quantidade de diretrizes e documentos (Hall *et al.*, 2022). Portanto, apesar das recentes

recomendações e orientações para implementação de técnicas de mudança de comportamento teoricamente informadas para prevenção secundária do AVE, possivelmente, isso não tem sido aplicado na pesquisa e na prática clínica em todo mundo. Além disso, essa revisão de escopo mostrou que, das 16 diretrizes e documentos identificados, 11 recomendaram a prática de atividade física (Hall *et al*., 2022). Porém, as orientações ainda são rasas, e não foi encontrado nenhuma diretriz para tratamento ou prevenção secundária do AVE que fornecesse orientações específicas para prescrição da atividade física no Brasil (Hall *et al*., 2022). Portanto, no contexto brasileiro, os indivíduos pós-AVE ainda devem receber orientações gerais para prática regular de atividade física, o que possivelmente não deve ser efetivo para resultar no aumento do nível de atividade física nessa população.

A Organização Pan-Americana de Saúde (OPAS) publicou recentemente um pacote técnico denominado HEARTS para manejo das doenças cardiovasculares, incluindo um documento específico contendo orientações para adoção de hábitos saudáveis, traduzido para o português-Brasil (OPAS, 2019). Este pacote técnico é constituído por seis módulos que correspondem a cada uma das letras da sigla HEARTS (OPAS, 2019). O módulo H, "hábitos saudáveis", que compreende aconselhamento ao paciente (OPAS, 2019). O módulo E, "Evidências", que compreende o uso de protocolos baseados em evidência (OPAS, 2019). O módulo A, "Acesso", que compreende acesso a medicamentos e tecnologias essenciais (OPAS, 2019). O módulo R, "Risco", que compreende manejo das doenças cardiovasculares baseado no risco (OPAS, 2019). O módulo "T", que compreende "Trabalho" de equipe como base para a atenção (OPAS, 2019). E, finalmente, o módulo "S", "Sistemas de monitoramento" (OPAS, 2019).

Nesse pacote técnico, os quatro principais fatores de risco comportamentais para doenças cardiovasculares são descritos: alimentação não saudável, tabagismo, consumo nocivo de álcool e atividade física insuficiente (OPAS, 2019). Para níveis saudáveis de atividade física para adultos, o pacote técnico HEARTS destaca a orientação de que todos os indivíduos, inclusive indivíduos pós-AVE, realizem, pelo menos: 150 minutos de atividade física moderada ou, pelo menos, 75 minutos de atividade física vigorosa, distribuídos durante a semana; ou uma combinação equivalente de atividade moderada e vigorosa; ou atividades de fortalecimento muscular, exercitando grandes grupos musculares em dois ou mais dias da semana (OPAS, 2019).

O pacote técnico HEARTS apresenta aspectos relacionados à mudança de comportamento e apresenta o modelo de intervenção breve usando a ferramenta 5As:

arguir, aconselhar, avaliar, assistir e acompanhar (OPAS, 2019). O uso desta ferramenta é recomendado para incentivar as mudanças de comportamento (OPAS, 2019). Considerando a taxonomia para técnicas de mudanças de comportamento proposta por Michie *et al*. (2013), o pacote técnico HEARTS aborda aquelas denominadas como metas e planejamento, *feedback* e monitoramento, e suporte social (OPAS, 2019). Até o momento, não foi identificado nenhum estudo que utilizou esse pacote técnico HEARTS para aumentar o nível de atividade física de indivíduos pós-AVE.

No Brasil, somente um ensaio clínico foi identificado no *Clinical Trials* tendo como objetivo a promoção de atividade física de indivíduos pós-AVE (Scianni, 2022). Esse ensaio clínico está sendo realizado com indivíduos pós-AVE na fase aguda e subaguda utilizando de estratégias teoricamente informadas (Scianni, 2022). Porém, diretrizes recomendam que a prática de atividade física deve ser um componente do cuidado contínuo de indivíduos pós-AVE (Billinger *et al*., 2014). Nesse sentido, torna-se necessário também promover a prática de atividade física com indivíduos pós-AVE na fase crônica, utilizando de estratégias teoricamente informadas. Além disso, não foi encontrado nenhum estudo que tenha utilizado o pacote técnico HEARTS com o objetivo de aumentar o nível de atividade física de indivíduos pós-AVE. Considerando que esse pacote técnico HEARTS foi traduzido para o português-Brasil, seu uso pode contribuir para o alcance de resultados significativos. Por fim, a literatura também tem se mostrado escassa quanto à promoção da prática de atividade física com indivíduos pós-AVE na fase crônica utilizando a combinação de estratégias, como por exemplo, o uso de pedômetros associado à outra estratégia (Lynch *et al*., 2018).

Além da identificação da necessidade do incentivo à prática de atividade física por indivíduos pós-AVE, é importante garantir que essa prática seja contínua (Billinger *et al*., 2014). Uma possível estratégia que pode contribuir para solucionar essa problemática é o uso da telessaúde. A telessaúde refere-se à prestação de serviços de reabilitação em um local remoto usando tecnologias de informação e comunicação (Brennan *et al*., 2009). O uso de intervenções por telessaúde tem sido adotado no cuidado à saúde de indivíduos pós-AVE e se tornou mais frequente com a pandemia da COVID-19 (Bloem *et al*., 2020). Intervenções teoricamente informadas para autogerenciamento de fatores de risco relacionados ao estilo de vida com indivíduos pós-AVE, como por exemplo, para aumentar o nível de atividade física (Lawrence *et al*., 2019), podem ser fornecidas remotamente por meio da telessaúde (Gladstone *et al*., 2022). No entanto, as recomendações para o uso de intervenções por telessaúde para mudança de

comportamento e autogerenciamento na prevenção secundária do AVE são baseadas em evidências classificadas como nível C (Gladstone *et al.*, 2022). Portanto, torna-se necessário investigar o efeito de intervenções aumentar o nível de atividade física de indivíduos pós-AVE por telessaúde para melhor recomendação da literatura quanto ao uso dessa intervenção.

1.1 OBJETIVOS

Objetivo primário: Investigar se uma intervenção teoricamente informada por telessaúde envolvendo o pacote técnico HEARTS e o uso de um monitor de atividade para aumentar o nível de atividade física é viável de ser implementada com indivíduos pós-AVE.

Objetivo secundário: Estimar os parâmetros para a realização de um ensaio clínico aleatorizado (ECA) considerando os desfechos nível de atividade física e número de indivíduos pós-AVE que se tornaram fisicamente ativos, pressão arterial sistólica (PAS) e diastólica (PAD), aptidão cardiorrespiratória, autoeficácia para a prática de atividade física e qualidade de vida relacionada à saúde.

1.2 Hipóteses do estudo

H0: Uma intervenção teoricamente informada por telessaúde envolvendo o pacote técnico HEARTS e o uso de um monitor de atividade para aumentar o nível de atividade física não é viável de ser implementada com indivíduos pós-AVE.

H1: Uma intervenção teoricamente informada por telessaúde envolvendo o pacote técnico HEARTS e o uso de um monitor de atividade para aumentar o nível de atividade física é viável de ser implementada com indivíduos pós-AVE.

2. METODOLOGIA

2.1 Delineamento

Trata-se de um estudo de viabilidade de fase 1 para condução de um ECA (Eldridge *et al.*, 2016), com avaliador cegado e com alocação oculta. O projeto será submetido ao Comitê de Ética em Pesquisa da Universidade Federal de Minas Gerais (UFMG). A folha de rosto encontra-se em anexo (ANEXO 1). Todas as anuências necessárias para o desenvolvimento do estudo foram obtidas (ANEXO 2). Além disso, esse projeto foi aprovado pela câmera do Departamento de Fisioterapia da UFMG (ANEXO 3).

Este estudo será registrado no www.ClinicalTrials.gov e realizado conforme as recomendações da extensão do CONSORT para estudos piloto ou de viabilidade (Eldridge *et al.*, 2016). O recrutamento dos indivíduos e a coleta de dados serão iniciados após a aprovação do Comitê de Ética em Pesquisa da UFMG.

Todos os indivíduos serão orientados sobre os procedimentos que serão realizados e apenas serão incluídos após concordarem com a participação voluntária e assinarem o Termo de Consentimento Livre e Esclarecido (TCLE) (APÊNDICE 1).

2.2 Participantes

Uma amostra de conveniência será recrutada na comunidade da cidade de Belo Horizonte, a partir de contato com ambulatórios, clínicas, hospitais, centros de saúde, associações, grupos de pesquisa e projetos de extensão desenvolvidos com a população de interesse.

Para a participação no estudo, os indivíduos deverão atender aos seguintes critérios de inclusão:

- Apresentar diagnóstico clínico de AVE há mais de seis meses;
- Ter idade igual ou superior a 18 anos;
- Ser classificado como "Inativo" de acordo com o Escore de Atividade Ajustado (EAA) do Perfil de Atividade Humana (PAH) (Souza *et al.*, 2006);
- Ser capaz de caminhar 10 metros de forma independente com ou sem dispositivo de auxílio de marcha (considerando que caminhar é uma forma potencial de participar de atividade física);
- Apresentar liberação médica para a prática de atividade física.

Serão excluídos indivíduos que apresentarem:

- Teste de rastreio positivo para possíveis alterações cognitivas (Mini-exame do Estado Mental) utilizando o ponto de corte baseado no nível de escolaridade (Bertolucci *et al*., 1994) e/ou afasia de compreensão, avaliada pela capacidade de responder, utilizando movimentos corporais, a comandos verbais ("levante o seu braço não comprometido e abra a sua mão") (Teixeira-Salmela *et al*., 2007);
- Presença de dor ou outra condição de saúde adversa que comprometa a realização do programa de intervenção proposto, como distúrbios vestibulares, artrite severa ou qualquer outro diagnóstico de doença do sistema nervoso.

2.3 Randomização e alocação oculta

Os participantes serão alocados em dois grupos (experimental e controle). A sequência de alocação será gerada por um *website* (www.randomization.com). Um assistente de pesquisa, que não estará envolvido no recrutamento, fará a aleatorização e colocará a sequência em envelopes opacos, numerados e selados. Os envelopes serão preparados antes do início do estudo por um assistente de pesquisa não envolvido na pesquisa. Cada participante será alocado em um dos grupos respeitando o conteúdo dentro dos envelopes. Para minimizar um possível viés decorrente do não mascaramento dos participantes, eles serão orientados a não comentar com o examinador sobre o grupo para o qual foram designados.

2.4 Intervenção

Os participantes serão submetidos a um programa de intervenção envolvendo o pacote técnico HEARTS (OPAS, 2019) e o grupo experimental terá a adição da prática de atividade física auto-monitorada com o uso do *Smartwatch Mi Band 7*®. Portanto, a única diferença entre os grupos é que os participantes do grupo controle não receberão o monitor de atividade física (*Smartwatch Mi Band 7*®). Para acompanhar a adesão ao protocolo, os indivíduos receberão um diário de prática regular de atividade física, o qual será preenchido, para cada dia de realização da atividade física, a duração e a pontuação subjetiva de esforço segundo a Escala Categórica Modificada de Borg (Borg, 1982, Cavallazzi *et al*., 2008) (APÊNDICE 2). O pesquisador responsável por implementar a intervenção (fisioterapeuta com experiência prévia em atendimento de indivíduos pós-AVE e um dos pesquisadores responsáveis pelo presente estudo) e um assistente de pesquisa (discentes de iniciação científica do curso de graduação em fisioterapia) serão treinados previamente para padronização da implementação dos protocolos de intervenção, o qual será realizado sempre pelo mesmo terapeuta. Um acompanhante/cuidador será instruído a auxiliar o indivíduo no preenchimento do diário, caso seja necessário.

2.4.1 Grupo experimental

Os participantes do grupo experimental realizarão um programa de intervenção envolvendo o pacote técnico HEARTS (OPAS, 2019) e a prática de atividade física auto-monitorada. O pacote técnico HEARTS apresenta o modelo para implementação de intervenção breve 5As para aumento da atividade física (OPAS, 2019). Esse método

envolve: arguir, aconselhar, avaliar, assistir, acompanhar (OPAS, 2019). Os participantes do grupo experimental receberão um monitor de atividade física (*Smartwatch Mi Band 7®*) (como orientado no tópico "assistir" no pacote técnico HEARTS) e serão acompanhados por telefone, uma vez por semana, visando identificar êxitos e dificuldades, bem como, para reforçar os êxitos e identificar soluções para as dificuldades identificadas (como orientado no tópico "acompanhar" no pacote técnico HEARTS) (OPAS, 2019). Esses indivíduos realizarão um programa de intervenção de 12 semanas. Na primeira semana, os indivíduos realizarão as quatro primeiras etapas da intervenção breve 5As em um encontro que será realizado presencialmente na casa do participante ou no Laboratório de Estudos em Reabilitação Neurológica do Adulto (NEUROLAB) na Escola de Educação Física, Fisioterapia, e Terapia Ocupacional (EEFFTO) (UFMG), segundo a disponibilidade do participante, onde eles serão orientados sobre como utilizar o monitor de atividade física (serão orientados a utilizá-lo durante o tempo em que estiverem acordados, por todos os dias, durante a realização do estudo), receberão o diário de prática regular de atividade física (apresentado na ficha de avaliação previamente desenvolvida, APÊNDICE 2), serão orientados sobre como preenchê-lo, e agendarão os melhores horários para recebimento da ligação telefônica semanal.

2.4.2 Grupo controle

Os participantes do grupo controle realizarão a mesma intervenção do grupo experimental, porém não receberão o monitor de atividade física (*Smartwatch Mi Band 7®*).

2.5 Mensuração dos desfechos

A mensuração dos desfechos primário e secundários será realizada no NEUROLAB. As mensurações serão realizadas na avaliação inicial (semana 0), imediatamente após o término do programa de intervenção (semana 12) e um mês após o término do programa de intervenção (semana 16). Na avaliação inicial (semana 0), serão coletados dados clínicos e demográficos de todos os indivíduos para fins de verificação dos critérios de elegibilidade, identificação e caracterização da amostra. Para isso, será utilizada uma ficha de avaliação previamente desenvolvida (APÊNDICE 2). Nas semanas 12 e 16, será utilizada uma ficha de reavaliação previamente desenvolvida semelhante à ficha de avaliação, além da adição de uma avaliação da satisfação do participante quanto à intervenção realizada (APÊNDICE 3). Esta coleta será realizada

por examinador previamente treinado, cegado para a alocação dos grupos. Todos os participantes serão instruídos a não comentar informações sobre o treinamento recebido.

2.5.1 Desfechos primários: medidas de viabilidade

Viabilidade do recrutamento

A viabilidade do recrutamento será determinada pela razão entre o número total de indivíduos elegíveis e o número total de indivíduos triados, e pela razão entre o número total de indivíduos elegíveis e o número total de indivíduos recrutados (Caetano *et al.*, 2023, Peniche *et al.*, 2022, Kim *et al.*, 2020, Regan *et al.*, 2021).

Viabilidade da intervenção

A viabilidade da intervenção será determinada examinando a retenção, acompanhamento dos indivíduos, atendimento/comparecimento, segurança e eficácia percebida.

A retenção será determinada pela razão entre o número total de indivíduos que completaram o programa de intervenção proposto e o número total de indivíduos que iniciaram o programa de intervenção proposto. Os motivos do abandono do programa de intervenção serão registrados (Peniche *et al.*, 2022, Kim *et al.*, 2020, Regan *et al.*, 2021, Hanlon *et al.*, 2022).

O acompanhamento dos indivíduos será determinado pela razão entre o número total de indivíduos que permaneceram no mesmo grupo ao qual foram inicialmente alocados até o *follow-u*p e o número total de indivíduos que foram alocados (Regan *et al.*, 2021).

O atendimento/comparecimento será determinado pela razão entre o número total de sessões realizadas e o número total de sessões ofertadas. O número e os motivos de ausência às sessões serão registrados (Caetano *et al.*, 2023, Peniche *et al.*, 2022, Regan *et al.*, 2021).

A segurança será determinada pelo número e motivos dos eventos adversos (por exemplo, dor, quedas, hospitalização e morte) identificados durante o período de intervenção e acompanhamento do indivíduo (Caetano *et al.*, 2023, Regan *et al.*, 2021, Hanlon *et al.*, 2022).

A eficácia percebida será determinada pela seguinte questão: “Comparando como era antes de você realizar a intervenção e agora, você acha que sua capacidade de realizar atividade física rotineira está: muito pior, moderadamente pior, um pouco pior, igual, um

pouco melhor, moderadamente melhor ou muito melhor?". A resposta fornecida será registrada pelo examinador independente.

Viabilidade da obtenção das medidas clínicas

A viabilidade da obtenção das medidas clínicas será determinada pela porcentagem dos desfechos clínicos medidos (relação entre o número de desfechos clínicos medidos e o número de desfechos clínicos propostos para serem medidos) e a porcentagem de participantes que preencheram o diário (relação entre o número de indivíduos que preencheram o diário e o número de indivíduos que iniciaram o programa de intervenção proposto) (Caetano *et al.,* 2023).

2.5.2 Desfechos secundários: medidas clínicas

Os desfechos clínicos serão avaliados para estimar os parâmetros para a realização de um ECA, incluindo: nível de atividade física e número de indivíduos pós-AVE que se tornaram fisicamente ativos, PAS e PAD, aptidão cardiorrespiratória, autoeficácia para a prática de atividade física e qualidade de vida relacionada à saúde.

O nível de atividade física e o número de indivíduos pós-AVE que se tornaram fisicamente ativos serão medidos com o PAH (Souza *et al.,* 2006). O PAH é um questionário, aplicado sob a forma de entrevista, constituído por 94 atividades, sendo cada uma graduada hierarquicamente de acordo com o equivalente metabólico requerido (Teixeira-Salmela *et al*., 2007, Souza *et al*., 2006). As atividades incluem cuidados pessoais, transporte, manutenção da casa, atividades sociais e de lazer e exercícios físicos. Para cada item, existem três respostas possíveis: "ainda faz a atividade", "parou de fazê-la" e "nunca a fez" (Teixeira-Salmela *et al*., 2007, Souza *et al*., 2006). O escore máximo de atividade (EMA) indica a atividade com o maior gasto energético que o indivíduo é capaz de realizar (Teixeira-Salmela *et al*., 2007, Souza *et al*., 2006). Um EAA é obtido subtraindo-se da pontuação máxima o número de atividades que o indivíduo parou de realizar e indica a média do nível de equivalente metabólico típico (Teixeira-Salmela *et al*., 2007, Souza *et al*., 2006). Pontuações mais altas indicam melhores resultados (Souza *et al.,* 2006). O nível de atividade física será operacionalizado pelo EAA, em pontos (Souza *et al.,* 2006). Outra medida do PAH é a "Classificação de Atividade", que fornece uma classificação global do nível de atividade do indivíduo em "Inativo", "Moderadamente ativo" e "Ativo". O número de indivíduos que se tornaram "Moderadamente ativo" e "Ativo" será identificado. O PAH é um instrumento já adaptado

para o Português-Brasil, que apresenta propriedades de medidas adequadas para a avaliação do nível de atividade física de indivíduos pós-AVE (Teixeira-Salmela *et al.*, 2007, Souza *et al.*, 2006).

PAS e PAD serão operacionalizadas como a média das pressões obtidas. Essas medidas serão obtidas utilizando o esfigmomanômetro aneroide da marca *Tycos*® (*WelchAllyn Inc.*, NY, USA, Modelo DS-44) e o estetoscópio (*Litmann Classic* II SE 3M®, USA).

A aptidão cardiorrespiratória será medida pelo PAH (Souza *et al.*, 2006). Uma das medidas do PAH é o "Consumo de Energia no Estilo de Vida", operacionalizado em $ml.kg^{-1}.min^{-1}$ (Souza *et al.*, 2006). O PAH é um instrumento válido para estimar a aptidão cardiorrespiratória de indivíduos pós-AVE (Brito *et al.*, 2022).

A autoeficácia para a prática de atividade física será operacionalizada considerando o escore obtido na Escala de autoeficácia para a prática de atividade física (Rech *et al.*, 2011). Não foi encontrado nenhum instrumento que apresente adequadas propriedade de medida para avaliar a autoeficácia para a prática de atividade física de indivíduos pós-AVE. A escala apresenta 10 questões, divididas em duas seções (Rech *et al.*, 2011). Cada questão deve ser respondida como sim (1 ponto) ou não (0 ponto) (Rech *et al.*, 2011). Os escores são computados com a soma das respostas de cada questão (Rech *et al.*, 2011). Quanto maior o valor, mais elevada é a autoeficácia para a prática de atividade física (Rech *et al.*, 2011). Rech *et al.* (2011) investigaram as propriedades de medida da escala e identificaram que ela apresenta validade, consistência interna e reprodutibilidade adequadas para avaliar a autoeficácia para a prática de atividade física de adultos brasileiros.

A qualidade de vida relacionada à saúde será operacionalizada considerando os escores obtidos na Escala de Qualidade de Vida Específica para o AVE (EQVE-AVE) (Ciconelli *et al.*, 1999, Lima *et al.*, 2008). A versão brasileira da EQVE-AVE possui doze domínios (energia, papel familiar, linguagem, mobilidade, humor, personalidade, autocuidado, papel social, raciocínio, função de membro superior, visão e trabalho/produtividade), totalizando 49 itens (Lima *et al.*, 2008). Existem três possibilidades de repostas, em uma escala de escore de cinco a um: quantidade de ajuda necessária para realizar tarefas específicas, quantidade de dificuldade experimentada quando é necessário realizar uma tarefa e grau de concordância com afirmações sobre funcionalidade (Lima *et al.*, 2008). O escore mínimo possível é 49 (pior percepção de qualidade de vida relacionada à saúde) e o escore máximo é 245 (melhor percepção de

qualidade de vida relacionada à saúde) (Lima *et al*., 2008). A referência para as respostas é a semana anterior (Lima *et al*., 2008). Esse instrumento é aplicado sob a forma de entrevista, pode ser administrado em pouco tempo e apresentam boa aplicabilidade clínica e adequadas propriedades de medida para avaliação desse desfecho de indivíduos pós-AVE (Ciconelli *et al*., 1999, Lima *et al*., 2008, Cabral *et al*., 2012). Esse instrumento já foi traduzido e adaptado para o Português-Brasil (Ciconelli *et al*., 1999, Lima *et al*., 2008).

2.6 Calculo amostral

Como estudo de viabilidade, nenhum cálculo formal do tamanho da amostra é realizado (Billingham *et al.*, 2013). Seguindo a recomendação proposta por Julious (2005) para estudos de viabilidade com grupos paralelos, o presente estudo apresentará um tamanho de amostra de 24 indivíduos, com 12 indivíduos em cada grupo.

2.7 Procedimentos

Durante a execução do estudo, as medidas de prevenção à disseminação do coronavírus serão observadas (COMITÊ PERMANENTE DE ENFRENTAMENTO DO NOVO CORONAVÍRUS DA UFMG, 2020; MINISTÉRIO DA SAÚDE, 2020). Os indivíduos serão agendados por horário objetivando evitar aglomerações. No momento do agendamento das avalições será realizada uma triagem para avaliar a presença de sintomas de síndrome gripal. Na presença de sintomas, o período de isolamento será respeitado, e o indivíduo será contatado novamente após o período de segurança. Os avaliadores irão utilizar máscaras cirúrgicas descartáveis. Será solicitado que os participantes também utilizem máscaras, e caso necessário, serão fornecidas máscaras de procedimento para uso durante a avaliação. Quando for necessário, a máscara poderá ser retirada para realização dos testes. Todos os equipamentos reutilizáveis serão devidamente higienizados e esterilizados. Por fim, será disponibilizado álcool em gel 70% para higienização das mãos (COMITÊ PERMANENTE DE ENFRENTAMENTO DO NOVO CORONAVÍRUS DA UFMG, 2020; MINISTÉRIO DA SAÚDE, 2020).

Os indivíduos interessados em participar voluntariamente do presente estudo serão avaliados para elegibilidade segundo os critérios descritos anteriormente. Aqueles que atenderem aos critérios estabelecidos serão alocados aleatoriamente em grupo experimental ou grupo controle. Todos os dados serão coletados por um mesmo avaliador, previamente treinado, com auxílio de um assistente de pesquisa, que serão mascarados

quanto à alocação dos participantes. Outro pesquisador com auxílio de um assistente de pesquisa será responsável pela administração da intervenção e serão mascarados em relação aos dados dos participantes.

Este estudo será realizado no NEUROLAB do Departamento de Fisioterapia da EEFFTO (UFMG).

2.8 Análise Estatística

Todas as análises estatísticas serão realizadas por um examinador independente, cegado quanto a todos os procedimentos realizados no estudo, inclusive com relação à alocação dos grupos. Estatísticas descritivas serão calculadas para todos os resultados. Os tamanhos dos efeitos serão calculados para determinar a magnitude das comparações dentro e entre grupos. Todas as análises serão realizadas com o programa estatístico SPSS 20.0 e será considerado um $\alpha=0,05$, ajustado para comparações múltiplas.

REFERÊNCIAS

Bertolucci PH, Brucki SM, Campacci SR, et al. O Mini-Exame do Estado Mental em uma população geral. Impacto da escolaridade. Arq Neuropsiquiatr. 1994;52(1):1-7.

Billinger S, Arena R, Bernhardt J, et al. Physical activity and exercise recommendations for stroke survivors: a statement for healthcare professionals from the American Heart Association/American Stroke Association. Stroke. 2014;45(8):2532-53.

Billingham SA, Whitehead AL, Julious SA. An audit of sample sizes for pilot and feasibility trials being undertaken in the United Kingdom registered in the United Kingdom Clinical Research Network database. BMC Med Res Methodol. 2013;13:104.

Bloem BR, Dorsey ER, Okun MS. The Coronavirus Disease 2019 Crisis as Catalyst for Telemedicine for Chronic Neurological Disorders. JAMA Neurol. 2020;77(8):927-28.

Borg GA. Psychophysical bases of perceived exertion. Med Sci Sports. 1982:14(5):377-81.

Brennan DM, Mawson S, Brownsell S. Telerehabilitation: enabling the remote delivery of healthcare, rehabilitation, and self management. Stud Health Technol Inform. 2009;145:231-48.

Bridgwood B, Lager KE, Mistri AK, et al. Interventions for improving modifiable risk factor control in the secondary prevention of stroke. Cochrane Database Syst Rev. 2018;5(5):CD009103.

Brito SAF, Aguiar LT, Quintino LF, et al. Assessment of V̇o(2peak) and Exercise Capacity After Stroke: A Validity Study of the Human Activity Profile Questionnaire. Arch Phys Med Rehabil. 2022;103(9):1771-6.

Cabral DL, Laurentino GE, Damascena CG, et al. Comparisons of the Nottingham Health Profile and the SF-36 health survey for the assessment of quality of life in individuals with chronic stroke. Rev Bras Fisioter. 2012;16(4):301-8.

Cadilhac DA, Prvu Bettger J. Health Policy and Health Services Delivery in the Era of COVID-19. Stroke. 2021;52(6):2177-79.

Caetano LC, Ada L, Romeu Vale S, et al. Self-management to promote physical activity after discharge from in-patient stroke rehabilitation: a feasibility study. Top Stroke Rehabil. 2023;30(1):32-42.

Carvalho-Pinto BP, Faria CD. Health, function and disability in stroke patients in the community. Braz J Phys Ther. 2016;20(4):355-66.

Cavallazzi TG L, Cavallazzi RS, Cavalcante YMC, et al. Avaliação do uso da Escala Modificada de Borg na crise asmática. Acta Paul Enferm. 2005;18(1):39-45.

Ciconelli RM, Ferraz MB, Santos W, et al. Tradução para a língua portuguesa e validação do questionário genérico de qualidade de vida SF-36 (Brasil SF-36). Rev Bras Reumatol. 1999;39(3):143-50.

COMITÊ PERMANENTE DE ENFRENTAMENTO DO NOVO CORONAVÍRUS DA UFMG, 2020. Protocolo de biossegurança e adequação do espaço físico na UFMG. 2020.

Craig P, Dieppe P, Macintyre S, et al. Developing and evaluating complex interventions: the new Medical Research Council guidance. BMJ 2008;337:a1655.

D'Isabella NT, Shkredova DA, Richardson JA, et al. Effects of exercise on cardiovascular risk factors following stroke or transient ischemic attack: a systematic review and meta-analysis. Clin Rehabil. 2017;31(12):1561–72

Damsbo AG, Kraglund KL, Buttenschøn HN, et al. Predictors for wellbeing and characteristics of mental health after stroke. J Affect Disord. 2020;264:358-64.

Eldridge SM, Chan CL, Campbell MJ, et al. CONSORT 2010 statement: extension to randomised pilot and feasibility trials. Bmj. 2016;355:i5239.

Eldridge SM, Lancaster GA, Campbell MJ, et al. Defining Feasibility and Pilot Studies in Preparation for Randomised Controlled Trials: Development of a Conceptual Framework. PLoS One. 2016;11(3):e0150205.

Feigin VL, Brainin M, Norrving B, et al. World Stroke Organization (WSO): Global Stroke Fact Sheet 2022. Int J Stroke. 2022;17(1):18-29.

Feigin VL, Stark BA, Johnson CO, et al. Global, regional, and national burden of stroke

and its risk factors, 1990-2019: a systematic analysis for the Global Burden of Disease Study 2019. Lancet Neurol. 2021;20(10):795-820.

Gittler M, Davis AM. Guidelines for Adult Stroke Rehabilitation and Recovery. JAMA. 2018;319(8):820-21.

Gladstone DJ, Lindsay MP, Douketis J, et al. Canadian Stroke Best Practice Recommendations: Secondary Prevention of Stroke Update 2020. Can J Neurol Sci. 2021:1-23.

Hall P, von Koch L, Wang X, et al. A Scoping Review of Non-Pharmacological, Non-Surgical Secondary Prevention Strategies in Ischaemic Stroke and TIA in National Stroke Guidelines and Clinical Audit Documents. Healthcare (Basel). 2022;10(3):481.

Hanlon SL, Bley BC, Silbernagel KG. Determining the feasibility of exercise therapy and activity modification for treating adolescents with heel pain: a study protocol. BMJ Open Sport Exerc Med. 2022;8(3):e001301.

Heron N, Kee F, Cardwell C, et al. Secondary prevention lifestyle interventions initiated within 90 days after TIA or 'minor' stroke: a systematic review and meta-analysis of rehabilitation programmes. Br J Gen Pract. 2017;67(654):e57-e66.

Julious SA. Sample size of 12 per group rule of thumb for a pilot study. Pharmaceut Statist. 2005;4(4):287–91.

Katsanos AH, Filippatou A, Manios E, et al. Blood Pressure Reduction and Secondary Stroke Prevention: A Systematic Review and Metaregression Analysis of Randomized Clinical Trials. Hypertension. 2017;69(1):171-79.

Kim BJ, Park JM, Park TH, et al. Remote blood pressure monitoring and behavioral intensification for stroke: A randomized controlled feasibility trial. PLoS One. 2020;15(3):e0229483.

Kleindorfer DO, Towfighi A, Chaturvedi S, et al. 2021 Guideline for the Prevention of Stroke in Patients With Stroke and Transient Ischemic Attack: A Guideline From the American Heart Association/American Stroke Association. Stroke. 2021;52(7):e364-e467.

Lager KE, Mistri AK, Khunti K, et al. Interventions for improving modifiable risk factor control in the secondary prevention of stroke. Cochrane Database Syst Rev. 2014;(5):CD009103.

Lawrence M, Asaba E, Duncan E, et al. Stroke secondary prevention, a non-surgical and non-pharmacological consensus definition: results of a Delphi study. BMC Res Notes. 2019;12(1):823.

Lawrence M, Pringle J, Kerr S, et al. Multimodal secondary prevention behavioral interventions for TIA and stroke: a systematic review and meta-analysis. PLoS One. 2015;10(3):e0120902.

Lennon O, Galvin R, Smith K, et al. Lifestyle interventions for secondary disease prevention in stroke and transient ischaemic attack: a systematic review. Eur J Prev Cardiol. 2014;21(8):1026-39.

Lennon O, Hall P, Blake C. Predictors of Adherence to Lifestyle Recommendations in Stroke Secondary Prevention. Int J Environ Res Public Health. 2021;18(9):4666.

Lima R, Teixeira-Salmela LF, Magalhaes L, et al. Propriedades da versão brasileira do stroke specific quality of life: aplicação do modelo Rasch. Rev Bras Fisioter. 2008;12(2):149-56.

Lin B, Zhang Z, Mei Y, et al. Cumulative risk of stroke recurrence over the last 10 years: a systematic review and meta-analysis. Neurol Sci. 2021;42(1):61-71.

Lopes MEM, Santos JM, Lima LAO, et al. PERFIL DE HÁBITOS SAUDÁVEIS DE INDIVÍDUOS PÓS ACIDENTE VASCULAR CEREBRAL E SEU CONHECIMENTO SOBRE ESTA CONDIÇÃO DE SAÚDE: RESULTADOS PRELIMINARES. Resumo: XXXI Semana de Iniciação Científica. Belo Horizonte: Universidade Federal de Minas Gerais, 2022.

Loprinzi P. Dose-response association of moderate-to-vigorous physical activity with cardiovascular biomarkers and all-cause mortality: considerations by individual sports, exercise and recreational physical activities. Prev Med. 2015;81:73–7.

Lynch EA, Jones TM, Simpson DB, et al. Activity monitors for increasing physical

activity in adult stroke survivors. Cochrane Database Syst Rev. 2018;7(7):CD012543.

Michie S, Richardson M, Johnston M, et al. The behavior change technique taxonomy (v1) of 93 hierarchically clustered techniques: building an international consensus for the reporting of behavior change interventions. Ann Behav Med. 2013;46(1):81-95.

Michie S, Wood CE, Johnston M, et al. Behaviour change techniques: the development and evaluation of a taxonomic method for reporting and describing behaviour change interventions (a suite of five studies involving consensus methods, randomised controlled trials and analysis of qualitative data). Southampton (UK): NIHR Journals Library, 2015.

Modrego PJ, Pina MA, Fraj MM, et al. Type, causes, and prognosis of stroke recurrence in the province of Teruel, Spain. A 5-year analysis. Neurol Sci. 2000;21(6):355-60.

Muller M. RECORRÊNCIA E LETALIDADE DO ACIDENTE VASCULAR CEREBRAL EM JOINVILLE, BRASIL: ESTUDO PROSPECTIVO DE BASE POPULACIONAL. Dissertação de mestrado. Joinville: Universidade da Região de Joinville, 2015. Available from: http://univille.edu.br/account/ppgsma/VirtualDisk.html?action=readFile&file=Dissertacao_Milena_Muller.pdf&current=/Dissertacoes_completas/2015.

Organização Pan-Americana da Saúde (OPAS). HEARTS Pacote de medidas técnicas para manejo da doença cardiovascular na atenção primária à saúde. Guia de implementação. Washington: Organização Pan-Americana da Saúde, 2019.

Peniche PC, Pinto APS, Ribeiro RLMN, et al. Recruitment, retention, attendance, and adherence of a randomized controlled trial to evaluate the effects of task-specific training with individuals post stroke. Fisioter Pesqui. 2022;29(1):22-8.

Prior PL, Hachinski V, Chan R, et al. Comprehensive cardiac rehabilitation for secondary prevention after transient ischemic attack or mild stroke: psychological profile and outcomes. J Mol Signal. 2017;37(6):428–36.

Rech CR, Sarabia TT, Fermino RC, et al. Propriedades psicométricas de uma escala de autoeficácia para a prática de atividade física em adultos brasileiros. Rev Panam Salud Publica. 2011:29(4):259–66.

Regan EW, Handlery R, Stewart JC, et al. Feasibility of integrating survivors of stroke into cardiac rehabilitation: A mixed methods pilot study. PLoS One. 2021;16(3):e0247178.

Ribeiro KSQS, Neves RF, Brito GEG, et al. PERFIL DE USUÁRIOS ACOMETIDOS POR ACIDENTE VASCULAR CEREBRAL ADSCRITOS À ESTRATÉGIA SAÚDE DA FAMÍLIA EM UMA CAPITAL DO NORDESTE DO BRASIL. RBCS. 2013;16:35-44.

Saunders D, Greig C, Mead G. Physical activity and exercise after stroke: Review of multiple meaningful benefits. Stroke. 2014;45:3742–47.

Scianni AA. Promoting Physical Activity After Stroke Via Self-management. ClinicalTrials.gov. 2022. Available from: https://clinicaltrials.gov/ct2/show/study/NCT05461976?cond=Stroke&cntry=BR&draw=2&rank=1.

Souza AC, Magalhães LC, Teixeira-Salmela LF. Adaptação transcultural e análise das propriedades psicométricas da versão brasileira do Perfil de Atividade Humana. Cad Saude Publica. 2006;22(12):2623-36.

Stroke Association, James Lind Alliance. Priorities in stroke rehabilitation and long-term care. 2021. Available from: https://www.stroke.org.uk/sites/default/files/research/priorities_in_stroke_rehabilitation_and_lon-term_care.pdf.

Teixeira-Salmela LF, Devaraj R, Olney SJ. Validation of the human activity profile in stroke: a comparison of observed, proxy and self-reported scores. Disabil Rehabil. 2007;29(19):1518-24.

Trabaquini D. CONSUMO ALIMENTAR DE PESSOAS APÓS SOFREREM ACIDENTE VASCULAR CEREBRAL. Trabalho de conclusão do curso (Enfermagem). Assis: Fundação Educacional do Município de Assis-FEMA, 2015. Available from: https://cepein.femanet.com.br/BDigital/arqTccs/1111370129.pdf.

Wang A, Wu L, Wang X, et al. Effect of recurrent stroke on poor functional outcome in transient ischemic attack or minor stroke. Int J Stroke. 2016;11(7):NP80.

Winstein CJ, Stein J, Arena R, et al. Guidelines for Adult Stroke Rehabilitation and Recovery: A Guideline for Healthcare Professionals From the American Heart Association/American Stroke Association. Stroke. 2016;47(6):e98-e169.

Zhong W, Geng N, Wang P, et al. Prevalence, causes and risk factors of hospital readmissions after acute stroke and transient ischemic attack: a systematic review and meta-analysis. Neurol Sci. 2016;37(8):1195-202.
